# Plant-Derived and Dietary Hydroxybenzoic Acids—A Comprehensive Study of Structural, Anti-/Pro-Oxidant, Lipophilic, Antimicrobial, and Cytotoxic Activity in MDA-MB-231 and MCF-7 Cell Lines

**DOI:** 10.3390/nu13093107

**Published:** 2021-09-04

**Authors:** Monika Kalinowska, Ewelina Gołębiewska, Grzegorz Świderski, Sylwia Męczyńska-Wielgosz, Hanna Lewandowska, Anna Pietryczuk, Adam Cudowski, Aleksander Astel, Renata Świsłocka, Mariola Samsonowicz, Anna Barbara Złowodzka, Waldemar Priebe, Włodzimierz Lewandowski

**Affiliations:** 1 Department of Chemistry, Biology and Biotechnology, Bialystok University of Technology, Wiejska 45E Street, 15-351 Bialystok, Poland; e.golebiewska@pb.edu.pl (E.G.); g.swiderski@pb.edu.pl (G.Ś.); r.swislocka@pb.edu.pl (R.Ś.); m.samsonowicz@pb.edu.pl (M.S.); 2 Institute of Nuclear Chemistry and Technology, 16 Dorodna Street, 03-195 Warsaw, Poland; s.meczynska@ichtj.waw.pl (S.M.-W.); h.lewandowska@ichtj.waw.pl (H.L.); 3 Department of Water Ecology, Faculty of Biology, University of Bialystok, Ciolkowskiego 1J Street, 15-245 Bialystok, Poland; annapiet@uwb.edu.pl (A.P.); cudad@uwb.edu.pl (A.C.); 4 Environmental Chemistry Research Unit, Institute of Biology and Earth Sciences, Pomeranian University in Słupsk, Arciszewskiego 22a Street, 76-200 Słupsk, Poland; aleksander.astel@apsl.edu.pl; 5 Faculty of Chemistry, Warsaw University of Technology, Noakowskiego 3 Street, 00-664 Warszawa, Poland; bzlowodzka@gmail.com; 6 Department of Experimental Therapeutics, The University of Texas MD Anderson Cancer Center, 1901 East Rd., Houston, TX 77054, USA; wpriebe@mac.com; 7 Institute of Agricultural and Food Biotechnology—State Research Institute, Rakowiecka 36 Street, 02-532 Warsaw, Poland; w-lewando@wp.pl

**Keywords:** hydroxybenzoic acids, antioxidant, lipophilic, antimicrobial, MCF-7, MDA-MB-231, spectroscopy, quantum chemical calculations, SAR

## Abstract

Seven derivatives of plant-derived hydroxybenzoic acid (HBA)—including 2,3-dihydroxybenzoic (2,3-DHB, pyrocatechuic), 2,4-dihydroxybenzoic (2,4-DHB, β-resorcylic), 2,5-dihydroxybenzoic (2,5-DHB, gentisic), 2,6-dihydroxybenzoic (2,6-DHB, γ-resorcylic acid), 3,4-dihydroxybenzoic (3,4-DHB, protocatechuic), 3,5-dihydroxybenzoic (3,5-DHB, α-resorcylic), and 3,4,5-trihydroxybenzoic (3,4,5-THB, gallic) acids—were studied for their structural and biological properties. Anti-/pro-oxidant properties were evaluated by using DPPH^•^ (2,2-diphenyl-1-picrylhydrazyl), ABTS^•+^ (2,2-azino-bis(3-ethylbenzothiazoline-6-sulfonic acid), FRAP (ferric-reducing antioxidant power), CUPRAC (cupric-reducing antioxidant power), and Trolox oxidation assays. Lipophilicity was estimated by means of experimental (HPLC) and theoretical methods. The antimicrobial activity against *Escherichia coli* (*E. coli*), *Pseudomonas aeruginosa* (*P. aeruginosa*), *Staphylococcus aureus* (*S. aureus*), *Bacillus subtilis* (*B. subtilis*), *Salmonella enteritidis* (*S. enteritidis*), and *Candida albicans* (*C. albicans*) was studied. The cytotoxicity of HBAs in MCF-7 and MDA-MB-231 cell lines was estimated. Moreover, the structure of HBAs was studied by means of experimental (FTIR, ^1^H, and ^13^C NMR) and quantum chemical DFT methods (the NBO and CHelpG charges, electrostatic potential maps, and electronic parameters based on the energy of HOMO and LUMO orbitals). The aromaticity of HBA was studied based on the calculated geometric and magnetic aromaticity indices (HOMA, Aj, BAC, I6, NICS). The biological activity of hydroxybenzoic acids was discussed in relation to their geometry, the electronic charge distribution in their molecules, their lipophilicity, and their acidity. Principal component analysis (PCA) was used in the statistical analysis of the obtained data and the discussion of the dependency between the structure and activity (SAR: structure–activity relationship) of HBAs. This work provides valuable information on the potential application of hydroxybenzoic acids as bioactive components in dietary supplements, functional foods, or even drugs.

## 1. Introduction

Free radicals are molecules, atoms, or ions that contain an odd (unpaired) number of electrons in the valence orbital. Therefore, they are described as short-lived, highly unstable, and extremely reactive chemical species [1,2,3]. Naturally occurring metabolic processes—especially those related to oxygen respiration—cause the formation of certain amounts of oxygen free radicals, such as superoxide anions (O_2_^•−^), hydrogen peroxide (H_2_O_2_), or a particularly harmful hydroxyl radical (HO^•^) [4,5]. Various lifestyle-related factors such as poor nutrition (e.g., dietary vitamin deficiencies or high-sugar diet), low physical activity, chronic stress, smoking, alcohol abuse, and air pollution can lead to excessive production of reactive oxygen species (ROS) in the human body [6]. A moderate ROS level in organisms has a positive effect on the immune system and cellular responses, while high concentrations can lead to cell dysfunction, changes in DNA structure, and even apoptosis [7]. Any imbalance between antioxidant defense and ROS production causes a phenomenon known as oxidative stress [8]; this process has been implicated in various degenerative diseases, such as cardiovascular diseases (e.g., coronary heart disease, atherosclerosis), autoimmune diseases (e.g., rheumatoid arthritis), neurodegenerative diseases (e.g., multiple sclerosis, Alzheimer’s disease, Parkinson’s disease), aging, and cancer (e.g., breast cancer) [9,10,11]. Providing antioxidants in food plays an important role in protecting the organism against oxidative stress [12].

**Figure 1:**
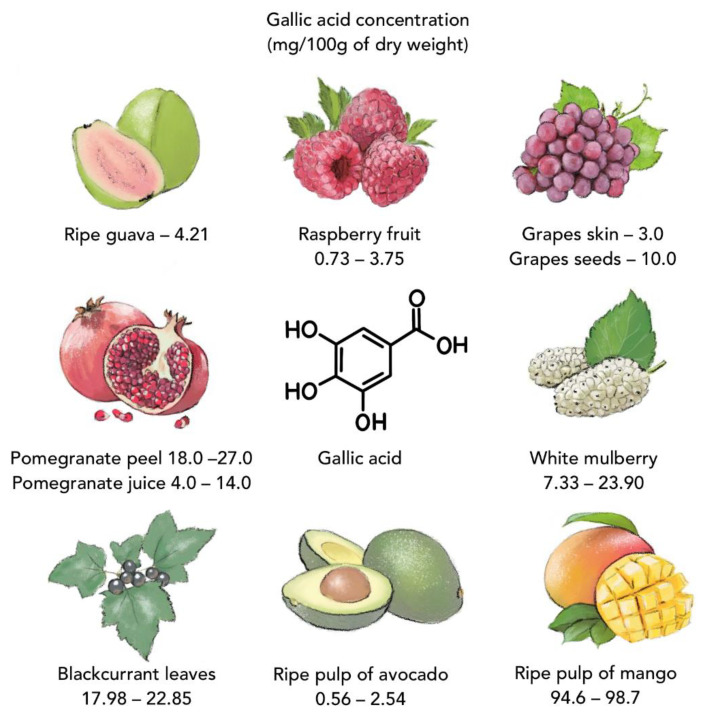
Some plants containing gallic acid (on the basis of data from [18,19,20,21,22,23,24,25,26,27,28]).

Fruits, vegetables, and herbs are natural, generally cheap, and widely available sources of many valuable nutrients (e.g., minerals, fibers, vitamins) and phytochemicals (e.g., flavonoids, phenolic compounds, anthocyanins) [13]. Phenolic compounds are secondary plant metabolites synthesized during biotic (caused by insects and microorganisms) and abiotic (caused by extreme temperatures, excessive lighting, excess water, drought, heavy metals, etc.) environmental stress [14,15]. Phenolic acids (i.e., benzoic acids and cinnamic acids) are a large subclass of phenolic compounds isolated from plants that possess extremely important biological properties, including antioxidant, anti-inflammatory, antiviral, antibacterial, antifungal, and anticancer properties. Epidemiological studies have revealed positive effects of plant phenolic compounds on human health, including reduced risk of cancer, asthma, allergy, and cardiometabolic disorders [16]. Because the human body cannot produce polyphenols, they must be obtained from food [17]. For example, bioactive gallic acid (3,4,5-THB), which belongs to the group of hydroxybenzoic acids, can be found in common plants such as guava (*Psidium guajava*) [18], grapes (*Vitis vinifera*) [19], pomegranate (*Punica granatum*) [20], avocado (*Persea americana*) [21], blackcurrant (*Ribes nigrum*) [22], mango (*Mangifera indica*) [23], and others (Figure 1) [24,25]. Another phenolic compound—gentisic acid (2,5-DHB)—occurs in citrus fruits (*Citrus* spp.), sesame (*Sesamum indicum*), olive (*Olea europaea*), rose gum (*Eucalyptus grandis*), and Jerusalem artichoke (*Helianthus tuberosus*). Protocatechuic acid (3,4-DHB) is found in onion, garlic, and relatives (*Allium* spp.), as well as mulberry (*Morus alba*), Japanese pepper (*Zanthoxylum piperitum*), mandarin (*Satsuma mandarin*), sharp-leaf galangal (*Alpinia oxyphylla*), and buckwheat (*Fagopyrum* spp.) [26,27,28].

The significant biological, pharmacological, and medicinal properties of plant phenolic compounds have been reported in many papers. For example, in the study of Kalinowska et al. [29], isoferulic acid was shown to exhibit slight anticancer activity against HaCaT human immortalized keratinocyte cell line [29]. Ashidate et al. [30] reported that gentisic acid (2,5-DHB) inhibited LDL (low-density lipoprotein) oxidation and the formation of cholesterol ester hydroperoxides in human plasma, which may play a huge role in the treatment of atherogenesis [30,31]. The study conducted by Shi et al. [32] showed that protocatechuic acid (3,4-DHB) isolated from the dried fruits of *Alpinia Oxyphylla Miq* may be a potential neuroprotective agent; the obtained results proved that 3,4-DHB increased the activity of glutathione peroxidase and superoxide dismutase, reduced the content of lipid peroxide, prevented the oxidative damage induced by hydrogen peroxide, and improved the cognitive properties of tested elderly rats [32]. It is well known that oxidative DNA damage can be responsible for the development of cancer [33,34]. In the study conducted by Lin et al. [35], protocatechuic acid (3,4-DHB) showed an apoptotic effect on human gastric adenocarcinoma (AGS) cells [35]. Lodovici et al. [36] reported a potent inhibitory effect on iron-induced oxidative DNA damage by 2,3-DHB and 3,4-THB [36]. In another study, 2,3-DHB decreased the level of NF-κB induction in monocytes treated with hydrogen peroxide. Data show that 200 μM 2,3-dihydroxybenzoic acid increased cell survival to 50%. These anti-inflammatory properties were related to the fact that 2,3-DHB can act as an antioxidant and an iron chelator [37]. Sharma et al. [38] reported that extract of *Hibiscus rosa-sinensis* containing gentisic acid (2,5-DHB) caused inhibition of the rate-limiting enzyme in tumor promotion (ornithine decarboxylase; ODC) [38].

The antioxidant and antiradical efficiency of hydroxybenzoic acids depends on various factors, one of which is the number and position of the hydroxyl (-OH) group attached to the aromatic ring [39]. It was reported that the antioxidant properties of the phenolic compounds increase with an increase in the number of -OH groups in the phenolic ring. For example, in a study by Brand-Williams et al. [40], gallic acid with three hydroxyl groups showed higher antiradical activity via DPPH^•^ assay than protocatechuic acid with two –OH groups [40]. The position of the hydroxyl groups attached to the benzene skeleton also affects the properties of the phenolic acid. In the study by Velika and Kron [41], dihydroxybenzoic acids with the –OH groups substituted in the *ortho* and *para* positions to the carboxylic (-COOH) group (2,5-DHB) showed stronger activity against superoxide radicals than phenolic acids with the -OH groups in the meta position with respect to one another (2,4-DHB; 2,6-DHB; 3,5-DHB) [41]. Although the antioxidant properties of selected group(s) of hydroxybenzoic acid have already been described in several other papers, the comprehensive analysis of the biological (antioxidant, anticancer, antimicrobial, lipophilic) and structural (based on spectroscopic and theoretical electronic parameters) properties of the group of seven hydroxybenzoic acids has not previously been conducted. Understanding the dependency between the chemical structure and biological activity of molecules is of great importance in designing new biologically active compounds that can be applied as functional food ingredients, dietary supplements, food antioxidants, preservatives, or drugs.

The present study aimed to examine the biological activity of seven selected hydroxybenzoic acids constituting a logical series of benzoic acid derivatives. The selected compounds differ with respect to the number and position of the hydroxyl groups substituted to the aromatic ring. The research evaluates the anti- and pro-oxidative potential, as well as the antimicrobial, cytotoxic, and lipophilic properties of the selected di- (and one tri-) hydroxy derivatives of benzoic acid. The methods used in this work include (1) the pro-/antioxidant tests, with the use of DPPH^•^ radicals, ABTS^•+^ cation radicals, ferric-reducing antioxidant power (FRAP), cupric-reducing antioxidant capacity (CUPRAC), and Trolox oxidation assays; (2) a microbiological microdilution method with the estimation of the minimum inhibitory concentration (MIC); (3) cytotoxicity testing in the MCF-7 and MDA-MB-231 human breast cancer cell lines; and (4) lipophilicity measurement via the use of HPLC. The structural studies were carried out using spectroscopic methods (FTIR, ^1^H, and ^13^C NMR) and quantum chemical calculations in the Gaussian 09W program; the electronic parameters ionization potential, electron affinity, electronegativity, chemical hardness, chemical softness, and electrophilicity index, as well as NBO and ESP atomic charge distribution, were calculated. Principal component analysis (PCA) was used in the statistical analysis of the obtained experimental and theoretical data.

## 2. Materials and Methods

### 2.1. Materials

2,3-Dihydroxybenzoic acid (2,3-DHB, pyrocatechuic acid), 2,5-dihydroxybenzoic acid (2,5-DHB, gentisic acid), 2,6-dihydroxybenzoic acid (2,6-DHB, γ-resorcylic acid), 3,5-dihydroxybenzoic acid (3,5-DHB, α-resorcylic acid), 3,4,5-trihydroxybenzoic acid (3,4,5-THB, gallic acid), DPPH (2,2-diphenyl-1-picrylhydrazyl), ABTS (2,2-azino-bis(3-ethylbenzothiazoline-6-sulfonic acid), potassium persulfate (K_2_S_2_O_8_), TPZT (2,4,6-tris(2-pyridil)-s-triazine), iron(III) chloride (FeCl_3_∙6H_2_O), sodium acetate (C_2_H_3_NaO_2_∙3H_2_O), copper(II) chloride (CuCl_2_), ammonium acetate (CH_3_COONH_4_), neocuproine (2,9-dimethyl-1,10-phenanthroline), iron(II) sulfate (FeSO_4_), Trolox (6-hydroxy-2,5,7,8-tetramethylchroman-2-carboxylic acid), hydrogen peroxide (H_2_O_2_), phosphate buffer pH 7, and horseradish peroxide (HRP) were purchased from Sigma-Aldrich Co. (St. Louis, MO, USA). 2,4-Dihydroxybenzoic acid (2,4-DHB, β-resorcylic acid) and 3,4-dihydroxybenzoic acid (3,4-DHB, protocatechuic acid) were purchased from Across Organics. Methanol was sourced from Merck (Darmstadt, Germany). All chemicals had an analytical purity and were used without further purification.

The following bacterial strains were used in this study: *Escherichia coli* (PCM 2857), *Pseudomonas aeruginosa* (PCM 2720), *Bacillus subtilis* (PCM 2850), *Staphylococcus aureus* (PCM 2267), *Salmonella enteritidis* (NCTC 4776), and *Candida albicans* (PCM 2566-FY). The microbial strains were bought from the Polish Collection of Microorganisms (Wroclaw, Poland) or the American Type Culture Collection. Bacteria (0.1 mL of the reconstituted suspension) were seeded onto sterile Mueller–Hinton agar plates (Biomaxima), to which previously suitable amounts of the tested compounds were added to give the desired concentration.

The human breast adenocarcinoma cell lines MDA-MB-231 and MCF-7 were purchased from the American Type Culture Collection (ATCC, Rockville, MD, USA) and maintained according to ATCC protocol. Briefly, MCF-7 cells were cultured in EMEM (Biological Industries, Israel) medium supplemented with 10% fetal calf serum (Gibco). MDA-MB-231 cells were cultured in Leibovitz‘s L15 (Biological Industries, Beit HaEmek, Israel) medium supplemented with 10% fetal calf serum (Gibco). The MCF-7 cells were incubated in a 5% CO_2_ atmosphere at 37 °C, whereas the MDA-231 cells were incubated in a 100% air atmosphere at 37 °C.

### 2.2. Methods 

**DPPH assay** was measured according to the method described by Rice-Evans [42]. Appropriate volumes of methanolic solutions of the tested hydroxybenzoic acids (C = 30 μM) were mixed with methanol in test tubes to prepare a series of solutions of various concentrations (final volume 1 mL). Then, 2 mL of methanolic solution of DPPH^•^ (C = 60 μM) was added to each tube; the control contained methanol instead of the sample. The tubes with reaction mixtures were capped, vortexed, and left to stand for 60 min at 23 °C in the dark. The absorbance of the solutions was measured by the use of a Cary 5000 UV–Vis spectrophotometer (Santa Clara, CA, USA) at λ = 516 nm against pure methanol. The ability to scavenge DPPH^•^ radicals (%I) was calculated according to the equation:

%I = [(A_control_ − A_sample_)/A_control_] × 100%

where %I = the % inhibition of DPPH^•^ radicals; A_control_ = the absorbance of the control; and A_sample_ = the absorbance of the tested sample. The concentration of the tested hydroxybenzoic acids was plotted against the %I, and the IC_50_ values (antioxidant concentration that inhibited 50% of DPPH^•^ radicals) were calculated from the obtained scavenging curves.

**ABTS assay** was conducted according to the procedure of Re et al. [43]. The reagent was prepared by mixing aqueous solutions of ABTS (C = 7 mM) and K_2_S_2_O_8_ (C = 2.45 mM) at a volumetric ratio of 1:1, and leaving them for 12–16 h at 23 °C to generate ABTS^•+^ cation radicals. Then, 1 mL of the obtained ABTS^•+^ solution was mixed with 60 mL of methanol. Samples containing 1 mL of ABTS^•+^ solution and 1 mL of methanolic solution of the tested compound (C = 50 μM) were mixed and incubated for 7 min at 23 °C; the control contained methanol instead of the sample. The absorbance of the solutions was measured with a UV–Vis spectrophotometer at 734 nm against methanol (the blank). The ability to scavenge ABTS^•+^ cation radicals was expressed as the %I of ABTS^•+^ and calculated according to the aforementioned equation. 

**FRAP assay** was carried out as described in [42]. The working FRAP reagent was prepared by mixing 250 mL of acetate buffer (C = 300 mM; pH 3.6), 25 mL of TPZT in 40 mM HCl (C = 10 mM), and 25 mL of FeCl_3_ aqueous solution (C = 20 mM) (10:1:1 *v*/*v*/*v*). Then, 3 mL of the freshly prepared FRAP reagent was added to 0.4 mL of methanolic solution of the tested compound (C = 50 μM) and mixed thoroughly. After 8 min of incubation at 23 °C, the absorbance at 595 nm was measured against the blank (containing 0.4 mL of methanol instead of the sample). Ferric-reducing antioxidant activity was expressed as Fe(II) ion equivalents (μM) using the calibration curve obtained for FeSO_4_ (the standard curve equation: y = 3.4303x − 0.1058; R^2^ = 0.9984).

**CUPRAC assay** was carried out according to the method described in [44]. First, 250 mL of CuCl_2_ (C = 10 mM; in water) was mixed with 250 mL of ammonium acetate (pH 7; in water) and neocuproine (C = 75 mM; in ethanol). Then, 3 mL of the CUPRAC mixture was added to 0.5 mL of methanolic solution of the tested compound (C = 50 μM) and 0.6 mL of distilled water. The samples were mixed and incubated for 60 min at room temperature (23 °C). The absorbance of the samples was measured with a UV–Vis spectrophotometer at λ = 450 nm against a blank (containing 0.5 mL of methanol instead of the sample). Cupric-reducing antioxidant activity was expressed as Trolox equivalents (μM) by using the calibration curve prepared over the range of 10–200 μM of Trolox (y = 4575.8x + 0.0271; R^2^ = 0.9919).

**The pro-oxidant activity** was expressed as the rate of Trolox oxidation according to the protocol described in [45]. First, a mixture of 0.5 mL of Trolox (C = 0.4 mM; in water) with 0.5 mL of H_2_O_2_ (C = 0.2 mM; in water), 0.5 mL of HRP (C = 0.04 μM; in phosphate buffer; pH = 7), 5 μL of the tested compounds (C = 50 μM; in methanol), and 495 μM of distilled water was prepared as the tested sample. The absorbance was measured at λ = 272 nm every 10 min for 60 min. The control contained methanol instead of the tested substance.

All anti-/pro-oxidant assays were conducted in five repetitions for three independent experiments for each tested compound. An Agilent Cary 5000 spectrophotometer (Agilent, Santa Clara, CA, USA) was used to measure the absorbance. The results were expressed as the means of the values obtained for the replications. Average, standard deviation calculation, and graphs were completed using Microsoft Excel 2019.

**The MIC** (minimal inhibitory concentration) was determined by serial dilution of the tested compound in an agar medium, to which the appropriate inoculum of microorganisms was then added and incubated. *Escherichia coli*, *Pseudomonas aeruginosa*, *Staphylococcus aureus*, *Bacillus subtilis*, *Salmonella enteritidis*, and *Candida albicans* were grown overnight and then resuspended in physiological saline to an optical density at 600 nm (OD = 600) of 0.60, corresponding to 5.0 × 10^8^ CFU/mL. Bacteria (0.1 mL of the reconstituted suspension) were seeded onto sterile Mueller–Hinton agar plates, to which previously suitable amounts of the tested compounds were added to reach the desired concentration. Tested chemicals were dissolved in DMSO solution. Negative controls were agar plates, to which DMSO was added, while positive controls were plates with gentamycin (in the case of bacteria) or flucanozole (in the case of fungi). The plates were incubated at 37 °C for 24 h. The lowest concentration without visible bacterial growth was determined as the MIC.

**Cytotoxicity evaluation** was conducted to assess the impact of hydroxybenzoic acids on the proliferation of MCF-7 and MDA-MB-231 cells, which was measured via neutral red (NR) assay. In brief, cells were seeded in 96-well microplates (TPP, Switzerland) at a density of 2 × 10^4^ cells/well in 100 μL of culture medium. At least three independent experiments in six replicate wells were conducted per concentration. Twenty-four hours after cell seeding, cells were treated with increasing concentrations of hydroxybenzoic acids (0.02–5 mM) for forty-eight hours. After treatment, cells were incubated for 3 h at 37 °C with 100 µL of neutral red solution (stock solution of NR) (Sigma-Aldrich), and then 5 mg/mL of PBS was diluted 1:100 in cell culture medium, incubated for 12 h at 37 °C, and centrifuged to remove any undissolved NR powder. Next, NR solution was aspirated, cells were washed with 150 µL of PBS, and 200 µL of an acid–ethanol solution (49% water, 50% ethanol, and 1% acetic acid) was added to each well. After 15 min of gentle shaking, optical density was read at 540 nm with an Infinite M200 plate reader spectrophotometer (Tecan, Austria). Based on the survival curve, the IC_50_ (the concentration of a compound that is required for the 50% inhibition of free radical viability) was calculated by fitting data to the logistic equation.

**The lipophilicity** of the studied compounds was determined via RP-HPLC analysis using a Waters Alliance 2695 HPLC separation module (Milford, MA, USA) and a Waters 2996 photodiode array detector (Milford, MA, USA) (λ = 254 nm). The analyses were carried out on the following chromatographic columns: RP-18e Purospher STAR (C18), 150 mm × 4.0 mm, 5 µm, (Merck, Darmstadt, Germany); RP-8e Purospher STAR (C8), 150 mm × 4.6 mm, 5 µm, (Merck, Darmstadt, Germany); Zorbax Eclipse XDB-CN (CN), 150 mm × 4.6 mm, 5 µm, (Agilent Technologies, Santa Clara, CA, USA); Nucleosil Phenyl (PHE), 250 mm × 4.6 mm, 7 µm, (Supelco, Bellefonte, PA, USA); and Regis Rexchrom IAM.PC.DD.2 (IAM), 100 mm × 4.6 mm, 10 µm, (Regis Technologies Inc., Morton Grave, IL, USA). The experimental conditions and the calculations were as described in [46]. 

### 2.3. Calculations in ACD/Labs and Gausssian09 Programs 

The values of the partition coefficients in the octan-1-ol/water solvent system and pKa were calculated using the ACD/Labs program [47].

The molecules of dihydroxybenzoic acids and trihydroxybenzoic (gallic) acid were optimized using the B3LYP/6-311++G(d,p) method in the gas phase, in water and methanol. Based on the obtained structural data (bond lengths), the aromaticity indices Aj [48], BAC [49], HOMA, GEO, EN [50], and Bird’s I6 index [51] were calculated on the basis of the equations described in the literature. The index values were normalized in such a way that for a pure aromatic system (benzene) they assumed a maximum value of 1 (Aj, BAC, HOMA index) [48,49,50] or 100 (Bird’s I6 index) [51], and for a non-aromatic system (cyclohexatriene) took values of 0. Using the GIAO method in B3LYP/6-311++G(d,p), the NICS magnetic indices [52] were calculated. The NICS index describes the value of the chemical shift of a given chemical compound calculated in the center of the aromatic ring [52]. The distribution of electronic charges on the atoms of the analyzed molecules was calculated using the NBO [53] and CHelpG [54] methods. The electrostatic potential (ESP) distribution maps were calculated using the CHelpG method. The electronic parameters ionization potential, electron affinity, electronegativity, chemical hardness, chemical softness, and electrophilicity index were calculated on the basis of the values of the energy of HOMO (highest occupied molecular orbital) and LUMO (lowest unoccupied molecular orbital). Theoretical calculations were performed using the Gaussian09 program [55]. 

### 2.4. FTIR, ^1^H, and ^13^C NMR Study 

The FTIR spectra were recorded with an Alfa (Bruker) spectrometer (Bremen, Germany) within the range of 400–4000 cm^−1^. Samples in the solid state were measured in KBr matrix pellets and using the ATR technique. The ^1^H and ^13^CNMR spectra were measured on a Bruker Avance II 400 spectrometer (Bremen, Germany) in DMSO solution at 25 °C. TMS was used as an internal reference. 

### 2.5. Statistical Analysis 

For overall exploration of the datasets including lipophilic (LogP_C18_, LogP_Galas_, LogP_exp_) and electronic parameters (pK_a1_, ΔE_(LUMO-HOMO)_, IP), aromaticity indices (BAC, HOMA, I6, NICS), FTIR (νC-(OH), β(CH) 18b, γ(CH) 11), and NMR (δC2, δC3) parameters as features designed to build the model—as well as the anti-/pro-oxidant and antimicrobial potential of the selected dihydroxybenzoates as accompanied features—principal component analysis (PCA) was used. PCA produces a low-dimensional representation of a dataset, finds a sequence of linear combinations of the variables that have maximal variance, and serves as an efficient tool for data visualization. Prior to its use, the impact of the implementation of the PCA on a dataset was checked using analysis of Pearson’s correlation matrix. The PCA itself was calculated in its default mode. 

## 3. Results

### 3.1. DPPH Assay 

**Figure 2:**
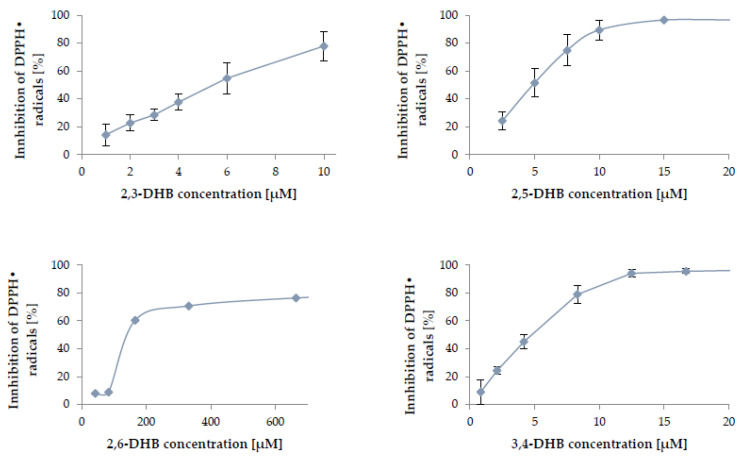

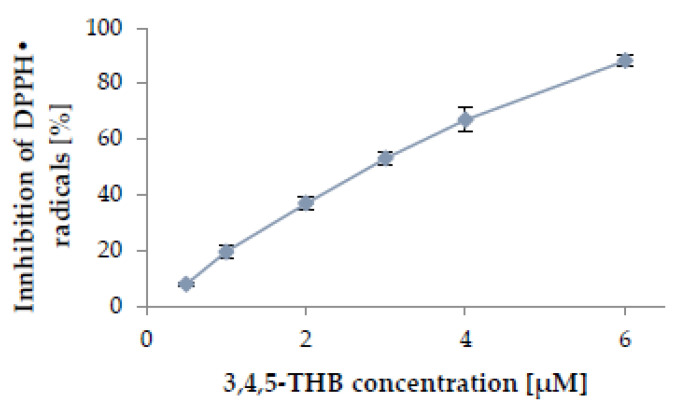
Scavenging of DPPH^•^ radicals by 2,3-DHB, 2,5-DHB, 2,6-DHB, 3,4-DHB, and 3,4,5-THB. Mean values from three independent experiments ± SD are shown.

To determine the antioxidant potential of the analyzed hydroxybenzoic acids to neutralize free radicals, one of the most popular and most frequently employed tests was used: the DPPH (2,2-diphenyl-1-picrylhydrazyl) assay. DPPH^•^ is a stable, synthetic free radical that has a free electron on the valence shell of the nitrogen atom that forms a nitrogen bridge [56]. Due to the delocalization of the unpaired electron, the DPPH^•^ molecule does not dimerize [57]. When the DPPH^•^ radical reacts with a compound capable of donating a hydrogen atom, it is reduced, and the solution turns from purple to yellow, as measured spectrophotometrically [58]. The antioxidant activity data for studied compounds, expressed as IC_50_ parameters (the concentration of antioxidant necessary to inactivate 50% of free radicals), are displayed in Table 1. The 2,4-DHB and 3,5-DHB showed the lowest antiradical properties (or no antiradical activity), with IC_50_ values higher than 120,000 and 1000 μM, respectively. Among the tested hydroxybenzoic acids, 3,4,5-THB—with three hydroxyl groups attached to the aromatic ring—possessed the highest DPPH^•^ radical scavenging activity (IC_50_ = 2.42 ± 0.08 μM). The inhibition of DPPH^•^ radicals increased with the increase in the concentration of the tested compound, as shown in Figure 2. The 2,3-DHB, 2,5-DHB, and 3,4-DHB showed a slightly lower ability to capture the radicals than 3,4,5-THB. The activity of the tested compounds can be ranked according to their increasing antioxidant potential, as follows: 2,4-DHB < 3,5-DHB < 2,6-DHB < 2,3-DHB < 3,4-DHB < 2,5-DHB < 3,4,5-THB.

The ability of the analyzed phenolic acids to capture the DPPH^•^ radicals depends on many factors, one of which is the number of hydroxyl groups in the structure and their position on the aromatic ring [59]. The compounds with the lowest antioxidant potential in this study were 2,4-, 3,5-, and 2,6-DHB. Due to the presence of two hydroxyl groups in the *meta*- position to one another, they showed negligible activity at the analyzed concentrations. The most effective polyphenols include those with a hydroxyl group in *para*- or *ortho*- positions towards one another [60]. Therefore, the fact that 3,4,5-THB shows the greatest activity towards the generated DPPH^•^ radicals is influenced by the presence of three hydroxyl groups in its structure, and by two of them being in the *ortho-* position in relation to the third one. Mishra et al. [61] also reported the high antioxidant activity of 3,4,5-THB in the DPPH^•^ assay. The IC_50_ parameter after 30 min of incubation with a free radical was 4.2 μM (which gave 0.07 μmol of antioxidant/μmol of DPPH^•^ versus 0.04 μmol of antioxidant/μmol of DPPH^•^ reported herein). In the above work, the activity of 3,4-DHB and 2,3-DHB was similar to the results obtained for 3,4,5-THB, due to the presence of hydroxyl groups in their structures in the *para*- and *ortho*- positions, respectively; this was also related to them being in the *ortho*- position to one another, as well as the presence of an internal hydrogen bond between the -OH substituents. This facilitates the transfer of hydrogen from the -OH group of the phenols to the radical [62]. 

A review of the available literature indicates that there are some papers describing the antioxidant activity of hydroxybenzoic acids (Table 2), but due to the different measurement procedures used (e.g., concentrations of reagents and samples, type of solvents, reaction time, temperature) it is difficult to compare the data obtained by different researchers.

### 3.2. ABTS Assay 

**Figure 3:**
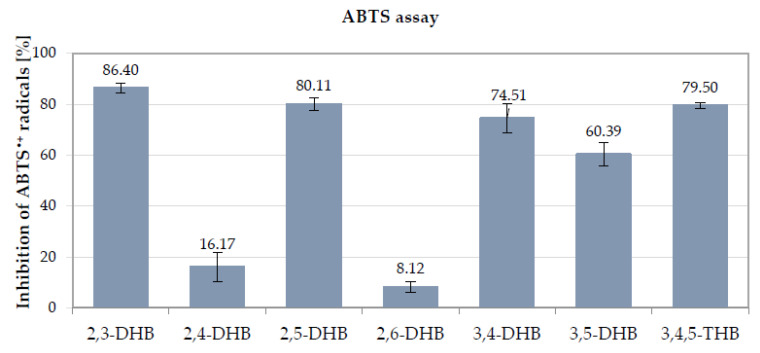
Comparison of the percentage of inhibition of ABTS^•+^ cation radicals measured after 7 min of reaction time for the tested hydroxybenzoic acids at a concentration of 50 μM (methanolic solutions). Mean values from three independent experiments ± SD are shown.

During the ABTS assay measurement, the freshly prepared 2,2-azino-bis(3-ethylbenzothiazoline-6-sulfonic acid (ABTS^•+^) cation radical was reduced by the antioxidant, which caused the discoloration of the blue-green solution and a reduction in the absorbance measured at 734 nm. The obtained results indicated that at the tested concentration (50 μM) the hydroxybenzoic acids showed low-to-significant scavenging properties against ABTS^•+^, as their average percentage of inhibition (%I) was over 60% (Figure 3). The highest antiradical activity was observed for 2,3-DHB, 2,5 -DHB, 3,4,5-THB, and 3,4-DHB, with %I values of 86.40, 80.11, 79.50, and 74.51%, respectively. These compounds also showed the best DPPH^•^ radical scavenging ability. 3,5-DHB was found to be less effective against ABTS^•+^ radicals, with %I = 60.39%, but much more active than 2,4-DHB and 2,6-DHB (16.17 and 8.12%, respectively). In contrast to the ABTS test, 3,5-DHB showed a negligible ability to capture the synthetic DPPH^•^ radicals.

### 3.3. FRAP Assay 

In the FRAP assay, the Fe(III)-2,4,6-tris(2-pyridil)-s-triazine (TPTZ) complex was reduced by the HBA to Fe(II)-TPTZ at an acidic pH (3.6), and the color of the solution changed to intense blue. The increase in the absorbance at 595 nm was measured. The obtained results are summarized in Table 3. Among all tested compounds, 2,5-DHB possessed the strongest FRAP antioxidant capacity (236.00 µM Fe^2+^). For 2,3-DHB and 3,4,5-THB, lower reduction potentials were reported (173.79 and 158.10 µM Fe^2+^, respectively). The FRAP values obtained for compounds at a concentration of 50 μM increased in the following order: 44.22 (3,4-DHB) < 158.10 (3,4,5-THB) < 173.79 (2,3-DHB) < 236.00 (2,5-DHB). The above list did not include other phenolic acids, due to the negative values of the FRAP units obtained.

### 3.4. CUPRAC Assay 

**Figure 4:**
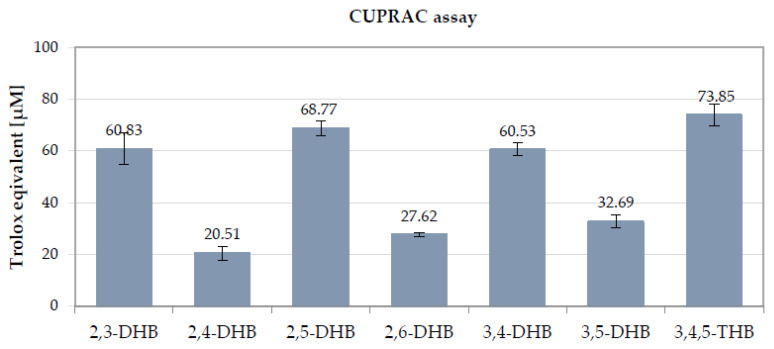
Comparison of the antioxidant activity of the tested hydroxybenzoic acids at a concentration of 50 μM (methanolic solutions), as measured by the CUPRAC assay. Mean values from three independent experiments ± SD are shown.

In this reducing assay, the light-blue Cu(II)-neocuproine complex was reduced by the antioxidants to yellow-orange Cu(I)-neocuproine at a close-to-physiological pH (7.4). The reaction was monitored at 450 nm. This method is based on the reduction of Cu(II) to Cu(I) by DHB and the formation of the yellow complex of Cu(I) with neocuproine (2,9-dimethyl-1,10-phenanthroline), which shows an absorption maximum at 450 nm [75]. The results of the above assay demonstrated that all tested compounds showed cupric-reducing antioxidant activity. The CUPRAC antioxidant capacity of the tested samples ranged from 20.51 to 73.85 μM of Trolox equivalents (Figure 4). The highest antioxidant capacity in the CUPRAC method was observed for 3,4,5-THB (73.85 μM of Trolox equivalents). Comparably high values were also obtained for 2,5-DHB, 2,3-DHB, and 3,4-DHB (68.77, 60.83, and 60.53 μM of Trolox equivalents, respectively). As in the DPPH, ABTS, and FRAP assays, these compounds were characterized by much higher antioxidant activity compared to the other tested compounds. 2,4-DHB, 2,6-DHB, and 3,5-DHB showed the lowest cupric-reducing antioxidant ability. According to these results, the total antioxidant capacity determined in CUPRAC assay follows the order 2,4-DHB < 2,6-DHB < 3,5-DHB< 3,4-DHB ~2,3-DHB < 2,5-DHB < 3,4,5-THB.

Summarizing the results of the four antioxidant assays based on different mechanisms of action (DPPH^•^, ABTS^•+^, FRAP, and CUPRAC assays), 2,3-DHB, 2,5-DHB, 3,4-DHB, and 3,4,5-THB showed the strongest antioxidant activity (Table 2 and Table 3, Figure 3 and Figure 4). The higher antioxidant activity of these DHBs can be explained by the resonance structures of their radicals [76]. In the case of DHBs with the -OH groups located in the *ortho-* and *para-* positions to one another, both oxygen atoms from the -OH groups participate in the delocalization of the electronic charge in the radical molecules. Meanwhile, the -OH substituents being in the *meta-* position of in the ring limits the delocalization of the charge with the participation of the second -OH [77]. 3,4,5-THB showed the highest antioxidant activity in the DPPH and CUPRAC assays; it contains the greatest number of hydroxyl (-OH) groups (three) bonded to the benzoic ring in an *ortho-* position to one another. It was previously shown that the antioxidant activity of phenolic compounds increases with the increasing number of hydroxyl groups substituted in the aromatic ring [39]. The same relationship was observed in the study of Sroka and Cisowski [69], where 3,4,5-THB showed a higher DPPH^•^ radical scavenging ability than 3,4-DHB and, 2,3-DHB which each contain two hydroxyl groups in their structures [69]. It should be stressed that, in our study, 3,4,5-DHB showed the highest antioxidant activity in the DPPH and CUPRAC assays, but not in the ABTS or FRAP assays. This may be related to differences in the mechanisms of action and reaction environments of the individual methods. The tests conducted in the above study can be divided into two types of reaction mechanisms: in the DPPH^•^ and ABTS^•+^ assays antioxidants can react with radicals by a combination of SET (single electron transfer) and HAT (hydrogen atom transfer) mechanisms (i.e., SPLET mechanism), whereas the FRAP and CUPRAC assays are based only on the SET reaction mechanism [57]. Moreover, the results of reactions based on the SET mechanism are related to the environment, e.g., the type of solvent and the pH value of the solution [78]. Other factors, such as the acidity of the studied molecules, may also affect the reaction pathway. The ABTS and FRAP assays are conducted predominantly in water solutions, where the hydroxybenzoic acids may be more ionized than in methanolic or ethanolic solutions. Phenoxide anions oxidize much more easily and faster than corresponding neutral molecules of phenols. This may accelerate the rate of electron transfer reaction in the SPLET mechanism. Moreover, the final results of ABTS assay may be affected by the reduction of the ABTS^•+^ by phenolic compounds to the parent substrate ABTS, the degradation of ABTS^•+^ to nonreactive products, or the formation of additional adducts between phenolics and ABTS^•+^ [79,80].

In conclusion, the experimental results show the following: (1) the antioxidant activity of the studied phenolic acids is directly related to the number and position of hydroxyl –OH groups bonded to the benzene ring; (2) hydroxybenzoic acids with hydroxyl groups substituted in the *ortho-* (2,3-DHB and 3,4-DHB) and *para-* (2,5-DHB) positions show higher antioxidant activity; (3) the hydroxyl groups substituted in the *meta-* (2,4-DHB and 3,5-DHB) position reduce the antioxidant and antiradical properties of hydroxybenzoic acids; and (4) the reaction environment and differences in the mechanisms of action of individual methods affect the activity of the compounds and the test results.

### 3.5. Pro-Oxidant Activity 

**Figure 5:**
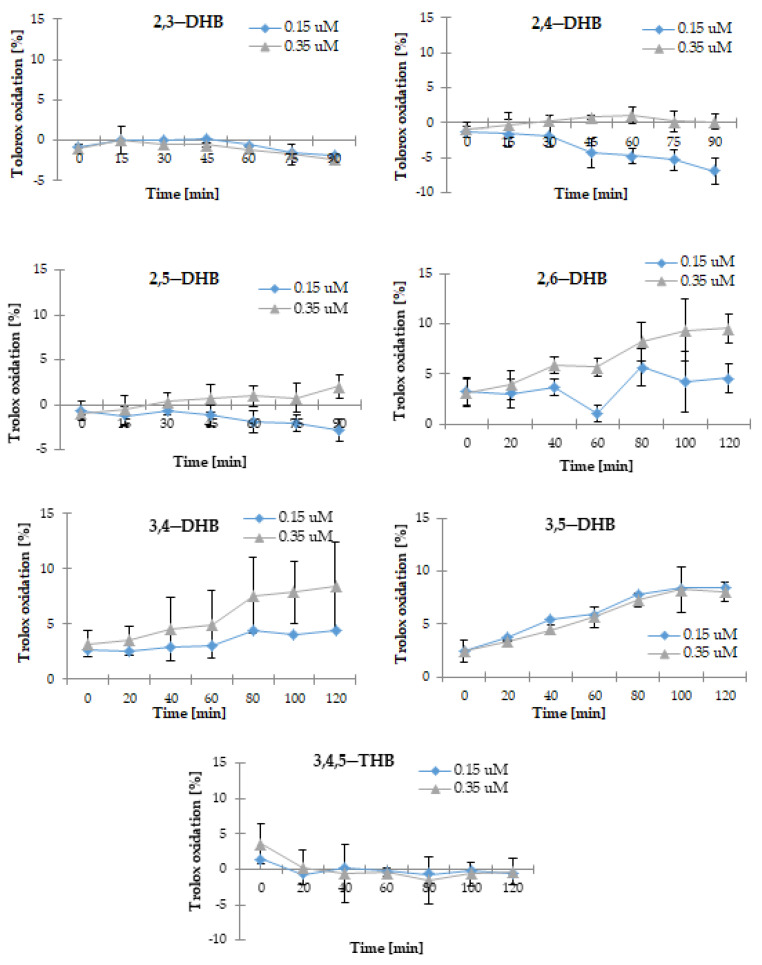
The degree of Trolox oxidation (%) by tested hydroxybenzoic acids at concentrations of 0.15 and 0.35 μM (methanolic solutions), measured within 90 or 120 min. Mean values from three independent experiments ± SD are shown.

HBAs were tested for their pro-oxidative effects in the Trolox oxidation assay. The radicals of HBAs were produced in their reaction with H_2_O_2_ catalyzed by horseradish peroxide. Then, the phenoxyl radicals react with Trolox, which undergoes oxidation to Trolox radicals, and then Trolox quinones, whereas the phenoxyl radicals are transformed to phenolic compounds. The maximum absorption for Trolox quinone is 272 nm. The pro-oxidant activity of the tested benzoic acid derivatives was measured at two concentrations (0.15 and 0.35 μM) and expressed as the rate of Trolox oxidation; the results are presented in Figure 5. 2,6-DHB, 3,4-DHB, and 3,5-DHB showed the highest pro-oxidant activity (degree of Trolox oxidation after 120 min of incubation for compounds at the concentrations of 35 μM: 9.52, 8.44, and 8.03%, respectively). The pro-oxidant activity of 2,4-DHB, 2,5-DHB, 2,6-DHB, and 3,4-DHB increased with an increase in their concentration; in the case of the other studied compounds, this cannot be clearly stated. The phenolic acids that possessed the strongest activity in the ABTS^•+^, FRAP, and CUPRAC assays (i.e., 2,3-DHB, 2,5-DHB, and 3,4,5-THB) showed the lowest pro-oxidant properties. Moreover, a low value of Trolox oxidation was obtained for 2,4-DHB.

### 3.6. Antimicrobial Activity

The antibacterial and antifungal activities of hydroxybenzoic acids were assayed using the microdilution method of minimum inhibitory concentration (MIC). The MIC values of the tested phenols for all of the tested strains were between 2 and 6 mg/mL (Table 4). The studies revealed that 2,4-DHB and 3,4-DHB possessed the strongest antimicrobial properties towards *E. coli*, *P. aeruginosa*, *S. aureus*, *B. subtilis*, *S. enteritidis*, and *C. albicans* at the concentration of 2 mg/mL. Similarly good results were obtained for 3,5-DHB, with only one higher MIC (3 mg/mL) for *P. aeruginosa*. 2,5-DHB, 2,6-DHB, and 3,4,5-THB presented the same antimicrobial activity against *E. coli*, *B. subtilis* and *S. enteritidis* at the concentration of 3 mg/mL. The lowest antimicrobial properties against the tested microorganisms were reported for 2,3-DHB (MIC = 5 mg/mL).

The antimicrobial activities of hydroxybenzoic acids have been widely described in previous research. In a study performed by Borges et al. [81], 3,4,5-THB (gallic acid) showed antimicrobial activity (MIC = 0.5–2.0 mg/mL) against *E. coli*, *P. aeruginosa*, *S. aureus*, and *L. monocytogenes* [81]. Sanchez-Maldonado et al. [82] obtained the MIC values for 3,4,5-THB and 3,4-DHB against *L. plantarum*, *L. hammesii*, *E. coli*, and *B. subtilis*, which ranged from 0.49 to 4.56 mg/mL for gallic acid and from 0.31 to 3.87 mg/mL for protocatechuic acid; these authors found that the increase in the number of -OH groups increased the growth-inhibitory activity of hydroxybenzoic acid, but this is not reflected in the results of our work [82]. In another study, 3,4-DHB and 3,4,5-THB isolated from *T. javanicum* was found to have significant activity against Gram-negative bacteria (*E. coli*, *P. aeruginosa*, *V. cholera*, *S. typhi*, and *S. typhimurium*) and some Gram-positive bacteria (*M. luteus* and *S. aureus*) [66]. Stojkovic et al. [83] reported the antibacterial activity of protocatechuic acid against *S. aureus*, *B. cereus*, *M. flavus*, *P. aeruginosa*, *E. coli*, and *E. cloacae*. The MIC value for all mentioned strains was 3.0 mg/mL [83]. 2,4-DHB (β-resorcylic acid) has been found to exhibit significant antimicrobial properties against various pathogens, including *Salmonella* [84], *E. coli* [85], and *L. monocytogenes* [86]. In the study of Jayesh et al. [87], 2,3-DHB electrospun into a nanofiber blend of poly(D,L-lactide) and poly(ethylene oxide) inhibited biofilm formation by *P. aeruginosa* [87]. Moreover, 2,3-DHB isolated from the fruit extract of *Flacourtia inermis* Roxb exhibited antifungal activity; at a concentration of 30 mg/mL it inhibited the growth of *A. fumigatus*, *A. flavus*, *A. niger*, and tested *Chrysosporium* species (zone of inhibition: 30, 24, 22, and 25 mm, respectively) [88]. Lattanzio et al. [89] reported that 2,5-DHB (gentisic acid) showed antifungal activity; at a concentration of 5 mM, it completely inhibited spore germination and mycelial growth of *Botrytis cinerea* and *Rhizopus stolonifer* [89]. In the work of Merkl et al. [30], 2,5-DHB inhibited the growth of *E. coli*, *B. aureus*, and *L. monocytogenes* (MIC = 2.5 mM) [90]. According to Campos et al. [91], the mechanism of the antimicrobial activity of phenolic acids may be related to their partially lipophilic character. It can be concluded that the protein denaturation is caused by an undissociated form of phenol that passes through the cell membrane via passive diffusion, disrupting its structure and possibly acidifying the cytoplasm [91].

### 3.7. Cytotoxicity Studies in MCF-7 and MDA-MB-231 Cell Lines

**Figure 6:**
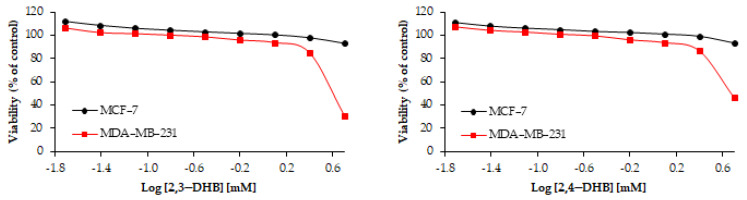

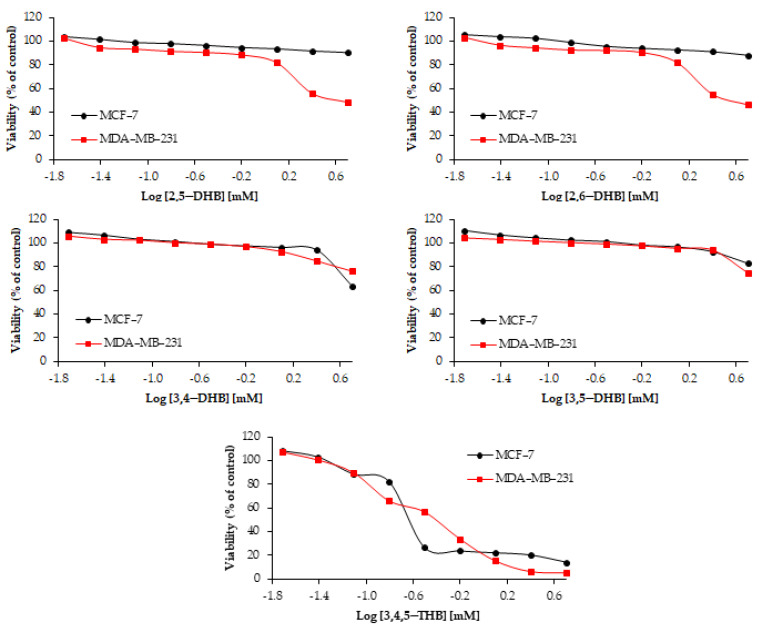
Survival curves of MDA-MB-231 and MCF-7 cells in the presence of different concentrations of the tested hydroxybenzoic acids.

The effects of seven hydroxybenzoic acids on two types of human breast cancer cell lines (MCF-7 and MDA-MB-231) were investigated; the results are shown in Table 5 and Figure 6. Among the tested compounds, only 3,4,5-THB (gallic acid) turned out to be toxic to the MCF-7 cell line. The IC_50_ parameter (concentration of the sample that inhibited cell viability by 50% compared to control cells) for gallic acid was 0.44 mM. The other tested hydroxybenzoic acids were found to be nontoxic to MCF-7 cells. 3,4,5-THB was found to be the most toxic among the tested compounds (IC_50_ = 0.36 mM) towards both MDA-MB-231 (estrogen-independent) and MCF-7 human breast cancer cells. At a concentration of 5 mM, 3,4,5-THB inhibited the growth of MCF-7 and MDA-MB-231 cells almost completely. Compared to the MCF-7 cell line, the MDA-MB-231 cell line turned out to be more sensitive to the tested hydroxybenzoic acids. Except for 3,4,5-THB, the studied compounds were only slightly toxic to the cancer cell lines. For example, the IC_50_ parameter for 2,4-DHB was 4.77 mM. 3,4-DHB and 3,5-DHB turned out to be nontoxic to both the MDA-MB-231 and MCF-7 cell lines. 

Numerous studies also indicate the antitumor potential of gallic acid. For example, in the study of Hsu et al. [92], 3,4,5-THB at a low concentration (5 μg/mL) significantly reduced the cell proliferation of MCF-7 to 42.73% of control (incubation time: 24 h) [92]. In another study [93], 3,4,5-THB was found to inhibit 50% of MCF-7 cancer cells at a concentration of 7.5 μg/mL (incubation time: 24 h) [93]. Maurya et al. [94] tested the effect of gallic acid on the A549 human lung adenocarcinoma cell line. Exposure to gallic acid for 24 h inhibited the A549 cell growth and decreased cell viability [94]. Moghtaderi et al. [95] reported that 3,4,5-THB inhibits the growth of MDA-MB-231 cells in a dose-dependent manner (IC_50_ ≈ 150 μM in 24 h); the combination of gallic acid with curcumin enhanced the apoptotic effect [95]. Another study [96] showed that 3,4,5-THB has anticancer activity against A2780 cancer cells (IC_50_ = 45.25 μg/mL; incubation time: two days), and this antiproliferative effect was greater for the synthesized derivatives of 3,4,5-THB (IC_50_ < 39 μg/mL) [96]. This fact may be related to the presence of alkyl chains, which increase the lipophilicity of the compound (easier transfer through the cell membrane) [97]. 

Other hydroxybenzoic acids that exhibited anticancer effects on the MDA-MB-231 cell line in our work were 2,3-DHB (IC_50_ = 4.10 mM) and 2,5-DHB (IC_50_ = 4.39 mM) (after 48 h of incubation); these compounds are often referred to as aspirin metabolites. Sankaranarayanan et al. [98] also reported their chemopreventive effects against that breast cancer line. 2,3-DHB was found to significantly inhibit the formation of colon cancer cells at a concentration of ~500 µM in MDA-MB-231 cells, whereas 2,5-DHB inhibited the colony at a concentration of 100 µM (incubated for 48 h) [98]. The effect of 2,5-DHB on MCF-7 cell proliferation was also examined in our previous study [62].

In our study, after 48 h of incubation, 3,4-DHB and 3,5-DHB did not show cytotoxic activity in the MCF-7 and MDA-MB-231 cell lines, but in the antimicrobial assay they possessed the strongest properties. In previous literature, protocatechuic acid has been referred to as a potent anticancer agent. In the study of Yin et al. [28], 3,4-DHB decreased the viability of MCF-7 human breast cancer cells, A549 lung cancer cells, HepG2 cells, HeLa cervical cancer cells, and LNCaP prostate cancer cells [28]. Yip et al. [99] reported that 3,4-DHB at a concentration of 100 µM inhibited HepG2 hepatocellular carcinoma cells’ viability to 40% (incubation time: 4 days) [99]. The results obtained by Reis et al. [100] showed that *Leccinum vulpinum* Watling extract—rich in protocatechuic acid (77 µg/g extract)—decreased cellular proliferation and induced apoptosis in MCF-7 human breast cancer cells [100]. 

### 3.8. Lipophilicity of Hydroxybenzoic Acids

**Figure 7:**
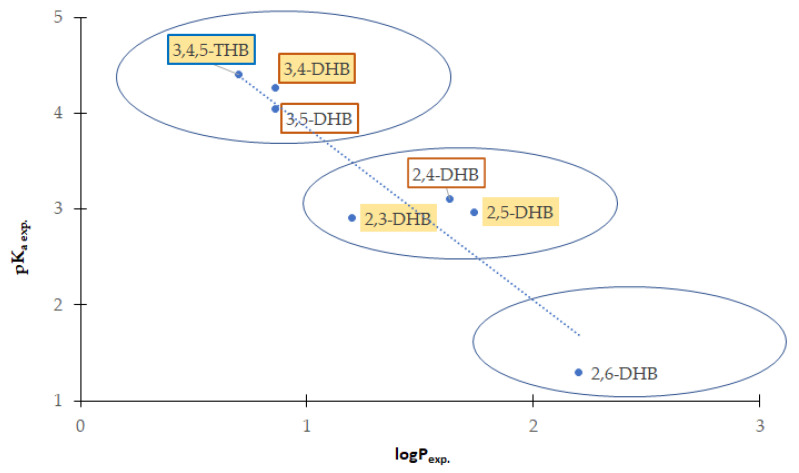
The dependency between the lipophilicity (logP_exp._) and acidity (pK_a exp._) in the series of studied hydroxybenzoic acids (yellow-marked: hydroxybenzoates with the highest antioxidant activity in the group; circled with a red square: compounds with the highest antimicrobial properties against selected microorganisms; circled with a blue square: compounds with the highest cytotoxic activity against selected cancer cell lines).

The chromatographic lipophilicity parameters (logk_w_) obtained under different chromatographic conditions are shown in Table 6. The alkyl (C18 and C8)-modified silica are stationary phases commonly applied for the estimation of lipophilicity by the use of HPLC, where the separation mechanism is mainly based on hydrophobic (van der Waals) interactions. In the case of the phenyl-modified silica phase (PHE), the π⟶π interactions are assumed. In the case of a more polar cyano-bonded phase (CN), the additional hydrogen bonds between cyano groups and a hydrogen atom from the hydroxyl group exist. The IAM (immobilized artificial membrane) stationary phase is used to mimic the distribution of compounds in the phospholipid membrane. The most distinct differences between particular values of log_kw_ were obtained for the IAM column, whereas the lowest differences were observed in the case of the CN and PHE columns. The highest linear correlation was obtained for the log_kw_ obtained by the use of the C18 and PHE columns (R^2^ = 0.772). The experimental logP values for HBAs (Table S1 and Table 7) highly correlate with the log_kw_ obtained on C18 and the theoretical logP classic, logP galas, pK_a1_, and pK_a2_ parameters. This means that both theoretical and chromatographic logP parameters can be successfully used to predict the hydrophilic–lipophilic properties of the hydroxybenzoic acid series. The obtained (experimental and theoretical) logP values for HBAs are in the range 0 < logP < 3; this means that these compounds possess from slightly hydrophilic (logP: 0–1) to moderately lipophilic properties (logP: 1–3) [101]. Generally, the disubstituted hydroxybenzoic acids were more lipophilic than the trisubstituted one (gallic acid has hydrophilic properties, and dissolves well in water and other polar solvents). According to the experimental logP values, the compounds can be ordered by increasing lipophilicity as follows: 3,4,5-THB→3,4-DHB~3,5-DHB→ 2,3-DHB→2,4-DHB →2,5-DHB →2,6-DHB. Dissociation and association phenomena affect the value of the partition coefficient. Therefore, we observed a relationship between the acid dissociation constants (expressed as pK_a_) and the values of partition coefficients. Each functional group that can be a hydrogen bond donor or acceptor increases the hydrophilic nature of the compound. Hydroxyl and carboxylic groups present in the structures of the compounds can form hydrogen bonds with water molecules in the aqueous environment, which affects their solubility in water. Therefore, in the case of 3,4,5-trihydroxybenzoic acid, an increase in the hydrophilic properties of the acid is noticeable. The more hydrogen bonds can be formed between a molecule and water molecules, the greater its solubility in water. In the case of ortho- substituted benzoates, the -OH group in the ortho- position is mostly engaged in hydrogen bond formation with the -COOH group, facilitating dissociation of the H^+^ carboxylic group and increasing the acidity of 2,6-DHB (pK_a_ = 1.30) and, to a lesser extent, 2,3-DHB (pK_a_ = 2.91), 2,5-DHB (pK_a_ = 2.97), and 2,4-DHB (pK_a_ = 3.11). In the case of 3,5-DHB (pK_a_ = 4.04), 3,4-DHB (pK_a_ = 4.26), and 3,4,5-THB (pK_a_ = 4.40), the -OH groups are too far from -COOH to interact with this functional group, but may form hydrogen bonds with the solvent. As a result, the acidity of these acids decreases (higher values of pK_a_) compared to the ortho-substituted benzoates. The dependency between acidity (pK_a exp._) and lipophilicity (logP_exp._) in the series of studied hydroxybenzoic acids is shown in Figure 7. With the increase in the acidity of the compounds, the lipophilicity increases as well. Therefore, the hydroxybenzoic acids can be divided into three groups characterized by: (a) lower acidity and lipophilicity (3,4,5-THB; 3,4-DHB; 3,5-DHB), (b) moderate acidity and lipophilicity (2,3-DHB; 2,4-DHB; 2,5-DHB), and (c) higher acidity and lipophilicity (2,6-DHB). There is no strict dependency between lipophilicity or acidity of hydroxybenzoic acids and their biological properties (antioxidant, antimicrobial, and cytotoxic), but general conclusions can be drawn. Dihydroxybenzoic acids with the highest antimicrobial activity are characterized by low-to-moderate lipophilicity (logP_exp._ in the range 0.86–1.63), and acidity at the level of pK_a_ 3.11–4.26 (Table 7). The strongest cytotoxic activity was observed in trihydroxybenzoic acid, with the lowest lipophilicity and acidity in the group of studied compounds.

### 3.9. DFT Study

The energy levels of the highest occupied molecular orbitals (E_HOMOs_) and the lowest unoccupied molecular orbitals (E_LUMOs_) for the optimized structures of the seven selected hydroxybenzoic acids were determined using the B3LYP/6-311++G(d,p) calculation method in the Gaussian 09W software. Using these data, other energy parameters such as HOMO–LUMO energy gap (ΔE), ionization potential (IP), electron affinity (A), electronegativity (χ), electrochemical potential (μ), electrophilicity index (ω), and chemical hardness (η) and softness (σ) were calculated, and the results are summarized in Table S2. The energy value of the HOMO orbital is a measure of the ability of one molecule to donate electrons to another, while the energy value of the LUMO orbital determines the tendency of the molecule to accept electrons. The lower the ionization potential of a compound (the minimum energy that is needed to detach an electron from the molecule) and the higher the energy of HOMO, the better the antioxidant (electron donor). The chemical activity of the substance also depends on other parameters, e.g., the value of the HOMO–LUMO energy gap [111,112]. The lower the value of ΔE, the easier the electron enters the excited state, and the better the antioxidant. Many physicochemical descriptors, such as those listed above, are often used to evaluate the biological and antioxidant activity of phenolic compounds [113]. According to the calculations (Table S2), we can see that the E_HOMO_ values for three phases did not show significant differences; the values of E_HOMO_ in the gas phase ranged from −9.2813 to −8.7735 eV, in aqueous solution from −9.2753 to −8.7555 eV, and in methanolic solution from −9.2755 to −8.7550 eV. Moreover, taking into account the energy of the HOMO orbitals, we can classify the studied compounds into two groups: group I—better electron-donor molecules (phenolic acids that presented the highest E_HOMO_ values, above −9 eV), i.e., 3,4,5-THB, 2,5-DHB, 2,3-DHB, and 3,4-DHB; and group II—better electron-acceptor molecules (phenolic acids that presented the lowest E_HOMO_ values, below −9 eV), i.e., 2,4-DHB, 3,5-DHB, and 2,6-DHB. In this work, the determination of the antioxidant activity of phenolic compounds was carried out in two environments: methanol (DPPH^•^) and water (ABTS^•+^, FRAP, CUPRAC). Hydroxybenzoic acids from group I showed better antioxidant activity in the above-mentioned assays than compounds from group II. According to the calculated parameters, 3,4,5-THB—with the highest energy of HOMO (-8.7550 and -8.7555 eV) and electronic chemical potential (−7.0637 and −7.0638 eV), and with the lowest values of ionization potential (8.7550 and 8.7555 eV) and electronegativity (−7.0637 and 7.0638 eV)—should be the best antioxidant among the tested phenolic acids (data for methanolic and aqueous solutions, respectively). In our study, gallic acid was found to have the highest antioxidant activity only in the DPPH^•^ and CUPRAC assays. Moreover, the relationship between the increase in antioxidant properties and the increase in the compound’s ability to donate electrons was observed only in the CUPRAC test. The energy values of the HOMO orbitals of hydroxybenzoic acids in the aqueous phase increased in the following order: 2,4-DHB (−9.2753 eV) < 2,6-DHB (−9.2475 eV) < 3,5-DHB (−9.2290 eV) < 3,4-DHB (−8.9340 eV) < 2,3-DHB (−8.9153 eV) < 2,5-DHB (−8.7749 eV) < 3,4,5-THB (−8.7555 eV).

### 3.10. Aromaticity

According to the values of the calculated geometric indices of hydroxybenzoic acids in which the hydroxyl groups were substituted in position 2,n-(where n = 3, 4, 5, 6), aromaticity increased in the sequence 2,3-DHB < 2,4-DHB < 2,5-DHB < 2,6-DHB (Table S3). This is indicated by the values of all indices calculated both for molecules modeled in the gas phase and in solvents (water, methanol). The values of the calculated aromaticity indices in water and methanol were very similar to one another. The aromaticity of the studied molecules increased with the change in the substitution site of the second carboxyl group (moving away) relative to the carboxyl group substituted in position 2. In the case of 3,4-DHB and 3,5-DHB, it was also observed that the molecules in which the hydroxyl groups were further apart had higher aromaticity. According to the calculated values of the aromaticity indices, among all tested systems, 3,5-DHB acid showed the highest aromaticity. High values of aromaticity indices were also noted for 3,4,5-trihydroxybenzoic acid (slightly lower than for 3,5-DHB and 2,6-DHB). When comparing the aromaticity of all studied molecules, they can be ordered by increasing aromaticity as follows: 2,3-DHB < 2,4-DHB < 2,5-DHB < 3,4-DHB < 2,6-DHB < 3,4,5-THB < 3,5-DHB. The NICS magnetic aromaticity index is negative for aromatic compounds; the lower its value, the higher the aromaticity of a given molecule. The highest aromaticity according to the NICS calculations was demonstrated for 3,4,5-trihydroxybenzoic acid, and the lowest for 2,4-DHB. The values were similar for calculations carried out in the gas phase and in solvents (water and methanol).

### 3.11. The Distribution of Electronic Charges

The electron charge distribution in the analyzed molecules was calculated using the CHelpG and NBO methods. The charge values are presented in Table S4. The electronic charge values correlate well with the values of chemical shifts of ^13^C NMR (presented in the next part of the manuscript). The positive electronic charges (NBO and CHelpG) were gathered around the carbon atom from the ring substituted by the -OH group (Figure S3). The highest electronic density was reported for the C1 carbon atom in 2,4- and 2,6-dihydroxybenzoic acids, dropped slightly for 2,3- and 2,5-dihydroxybenzoic acids, and was much lower for 3,4- and 3,5-dihydroxybenzoic acids and 3,4,5-trihydroxybenzoic acid. The electronic density around the second carbon atom became positive due to the pulling out of electrons by the highly electronegative oxygen atom of the hydroxyl group in 2,n-dihydroxybenzoic acids, while in the remaining acids these values were negative. The electronic density around the sixth atom was negative in almost all acids, except for 2,6-dihydroxybenzoic acid. In the case of the carbon of the carboxyl group (C7), the electronic density was positive in all analyzed acids, while for 2,6-dihydroxybenzoic acid it was much lower than in the other structures. This acid is characterized by high values of aromaticity indices, similar to 3,5-dihydroxybenzoic acid, in which the value of electronic density around the carbon atom of the carboxylic group is also lower than in other molecules. 

The symmetric position of the hydroxyl groups in the aromatic ring with respect to the carboxylic group significantly influences the decrease in the electronic density of the carbon atom of the carboxylic group, as well as the increase in symmetrization of the electronic charge distribution in the aromatic ring and, consequently, the increase in the aromaticity of a given system. Moreover, the calculated NBO and CHelpG total electronic charges of the ring (∑ē ring) increase in the order 2,6- < 3,5- < 2,4- < 2,3- < 3,4- < 2,5-DHB < 3,4,5-THB. For all dihydroxybenzoates, the value of the ∑ē ring was negative, whereas for 3,4,5-THB the total electronic charge of the ring had a positive value. Surprisingly, the increase in the ∑ē ring correlates well with the increase in the antioxidant activity of the studied molecules (specifically, it is in line with the results of the DPPH and CUPRAC assays). For 2,6-DHB (the molecule with the lowest antioxidant activity), the lowest electronic charge in the ring and the highest electronic charge in the COO^−^ were reported. The NBO charges calculated for the oxygen of the -OH group for 3,4,5-THB and 2,3-, 2,5-, and 3,4-DHB were more negative than for the other DHBs, facilitating the donation of protons in the reaction with radicals (the ionization potential also points to easier electron donation by 3,4,5-THB and 2,3-, 2,5-, and 3,4-DHB; Table S2).

### 3.12. ESP Maps

**Figure 8:**
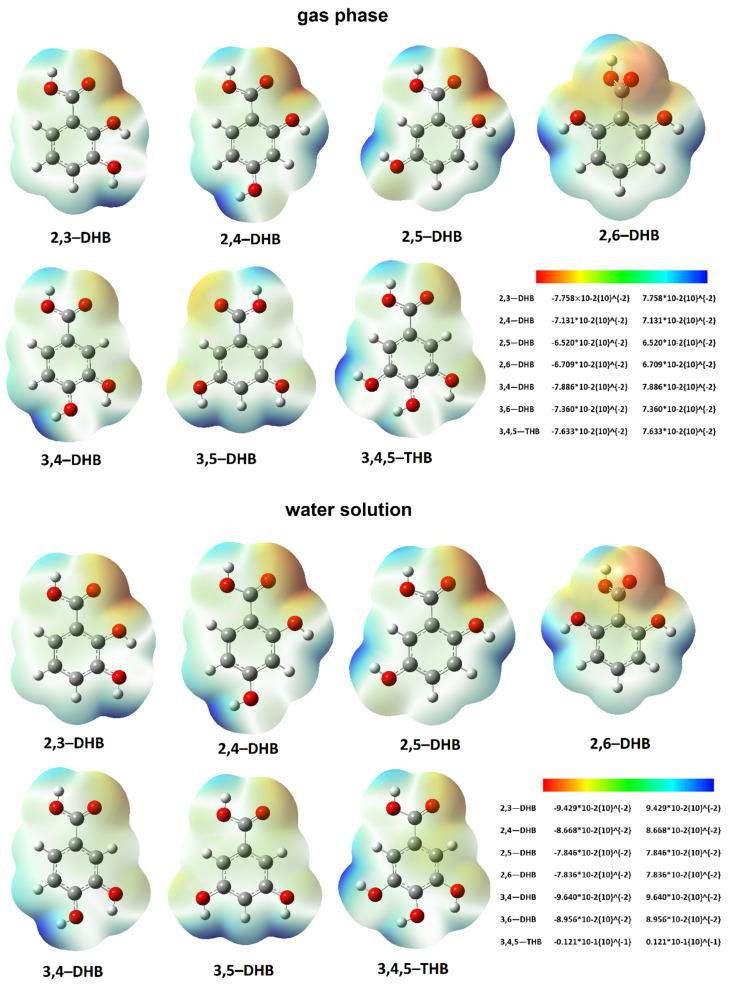
Electrostatic potential maps (calculated via the CHelpG method in B3LYP/6-311++G(d,p) for dihydroxybenzoic acids and 3,4,5-trihydroxybenzoic acid (in the gas phase and water solution).

The electrostatic potential map shows the areas of a molecule related to its electrophilic (red) and nucleophilic (blue) reactivity (Figure 8). In 2,3-, 2,4-, 2,5-, and 2,6-dihydroxybenzoic acids, it was observed that the oxygen atom of the carbonyl group was highly susceptible to electrophilic attack. As the second hydroxyl group moves away from the substitution position of the first hydroxyl group (the second position on the aromatic ring), the charge density in the region of the carboxylic group increases. Moreover, the area of the oxygen atom of the hydroxyl group (from the second position) is characterized by high electrophilic reactivity. The second position of the hydroxyl group in these acids is characterized by a significant susceptibility to nucleophilic attack, and as this hydroxyl group is moved away from the position of the first hydroxyl group, its nucleophilic reactivity increases. In 3,4-DHB, 3,5-DHB, and 3,4,5-THB, the electrophilic reactivity of the carbonyl group was lower than in the remaining acids. Moreover, the electrophilic reactivity of the hydroxyl group located closer to the carbonyl group was lower than in the other acids. In molecules with a symmetrical distribution of hydroxyl groups in the aromatic ring with respect to the carboxyl group—i.e., in 2,6-DHB and 3,5-DHB—a symmetric distribution of electrostatic potential was observed. These molecules are characterized by the highest aromaticity among the tested compounds. The distribution of the electrostatic potential for the molecules optimized in water and methanol was analogous to that in the molecules tested in the gas phase.

### 3.13. FTIR, ^1^H, and ^13^C NMR Study

The wavenumbers, intensities, and assignments of the bands occurring in the FTIR spectra of dihydroxybenzoic acids and 3,4,5-trihydroxybenzoic acid are presented in Table S5 [114]. The bands were numbered along with the notation used by Varsányi [115]. In the spectra of hydroxybenzoic acids (Figure S1), the bands assigned to the stretching vibrations of the carbonyl group νC=O were located in the range of 1639–1704 cm^−1^. These were very intense and characteristic bands, whose location in the spectra was affected by the number and position of hydroxyl substituents in the ring. In the range of 3214–3375 cm^−1^, the bands derived from -OH stretching occurred. The narrow band at 3374 cm^−1^ corresponded to the free, unassociated -OH group present only in the spectra of 2,4-DHB. The hydrogen atom of the -OH group can easily form intramolecular hydrogen bonds with the oxygen atoms of other functional groups, which affects the shape and position of the absorption bands assigned to the -OH stretching vibrations; this causes widening of the bands. The formation of hydrogen bonds leads to the formation of dimers and polyassociates. The spectra of carboxylic acids were characterized by very wide bands (with 3–5 maxima) assigned to the stretching of the -OH group (related to the presence of intermolecular hydrogen bonds), the maxima of which were in the range of 2500–2878 cm^−1^. In the spectra of all studied hydroxybenzoic acids, strong bands assigned to the stretching vibrations of νC-OH occurred in the range of 1194–1263 cm^−1^, and deformations of the -OH group at 1385–1446, 1208–1241 (in-plane β(OH)), and 576–633 (out-of-plane ν(OH)). Based on the FTIR spectra, the stabilization/destabilization of the aromatic system of ligands can be discussed. The destabilization of the aromatic system occurs via a decrease in the wavenumbers as well as the number and intensities of the bands assigned to the vibrations of the aromatic ring [116,117]. In the spectra of the 2,n-dihydroxybenzoic acids (n = 3,4,5,6), the increase in the wavenumbers of selected bands derived from the vibrations of the aromatic ring (no. 8a, 19a, 14, 18b, 7b, 9b) was observed in the following sequence: 2,3-DHB < 2,4-DHB < 2,5-DHB < 2,6-DHB. This may show the increase in the aromaticity of the acids in the same sequence (the calculation of the aromaticity indices confirmed the observation). The analysis of the bands assigned to the aromatic ring vibrations in the spectra of 3,n-dihydroxybenzoic acids (n = 4), revealed that 3,5-DHB showed higher aromaticity than 3,4-DHB. This can be concluded based on the higher wavenumber values of the aromatic bands and the occurrence of some additional aromatic bands in the spectrum of 3,5-DHB compared with the spectrum of 3,4-DHB. The highest wavenumber values of some aromatic bands were observed in the spectrum of 2,6-DHB, compared with other hydroxybenzoic acids, which may suggest the highest stability of the aromatic system in the case of this acid.

The change in the positions of signals from the ^1^H and ^13^C NMR spectra of hydroxybenzoic acids (Figure S2) showed that the change of the substituted position in the ring affects the electronic charge distribution in the molecule and the aromaticity of the system (Table S6). Strongly electronegative oxygen atoms of the -OH groups decrease the π-electron density at the nucleus, deshielding the nucleus and causing a larger chemical shift in the ^13^C NMR. The values of the chemical shifts of the carbon atoms substituted by the -OH group are highlighted in bold in Table S6. The highest decrease in the electronic charge was observed in the case of the second and fourth carbon atoms in 2,4-DHB, the second and fifth in 2,5-DHB, and the third and fourth in 3,4-DHB. These acids may reveal a greater ability to donate electrons or protons to radical molecules and, therefore, may show higher antioxidant activity than the other hydroxybenzoic acids.

The number and position of the -OH substituents in the ring affect the -COOH substitution capability by metal cations, as well as the strength of the hydroxybenzoic acids. 3,4- and 3,5-DHB, as well as 3,4,5-THB, are weaker acids than the other studied compounds. 

The electronic density around the C7 atom in 3,4- and 3,5-DHB, as well as 3,4,5-THB, is higher than in the other acids. Significant differences in the electronic density values around the C1 carbon atom were observed between the 2,n-dihydroxybenzoic acids and the 3,n-dihydroxybenzoic acids. In the case of the former, the electronic density was much higher (lower values of the chemical shifts in the ^13^C NMR spectra) than in the latter group of acids, as a result of the substitution site of the hydroxyl groups. As the position of the hydroxyl groups moves away from the carboxyl group, the electronic density around the carbon atom of the carboxyl group decreases (the value of ^13^C chemical shifts increases).

The comparison of the values of chemical shifts on the ^13^C NMR spectra (differences between the maximum value of the chemical shift and the lowest value of the chemical shift within a given molecule) of the tested acids shows that the most stable distribution of electronic charge occurs in the aromatic ring of 2,5-dihydroxybenzoic acid. The least stable electron charge distribution of the aromatic ring was observed in 2,6- and 3,5-dihydroxybenzoic acids. The values of chemical shifts were compared with the electronic charge distribution calculated by the NBO method. For electron densities determined theoretically by the NBO method and the experimental values of ^13^C on the C1, C2, C5, and C6 atoms, a high correlation R > 0.9 was noted.

The electronic stability of the aromatic ring (aromaticity of the π-electron system under study) can be assessed by analyzing the values of chemical shifts of aromatic protons in the ^1^H NMR spectra. Along with the change in the position of the hydroxyl groups, a change in the value of the chemical shifts of the protons attached to the aromatic ring was observed. The increase in the value of chemical shifts of aromatic protons in the ^1^H NMR spectrum indicates an increase in the aromaticity of the benzene ring. In the case of the tested acids, due to the different sites of substitution of the hydroxyl groups in the ring, the assessment of aromaticity of these compounds based on the criterion of the value of protic chemical shifts is ambiguous.

Changes in the values of chemical shifts of the proton of the carboxyl group and the protons of the hydroxyl groups in individual acids were also observed. The highest values of chemical shifts of hydroxyl groups were observed in the ^1^H NMR spectrum of 2,4-dihydroxybenzoic acid, and the lowest for 2,5-dihydroxybenzoic acid. The electronic density around the protons of the hydroxyl groups according to the values of the proton chemical shifts observed in the ^1^H NMR spectra increases as follows: 2,6-DHB < 2,4-DHB < 3,5-DHB < 3,4-DHB < 2,3-DHB < 2,5-DHB < 3,4,5-THB. The highest electronic density was observed around the protons of the hydroxyl groups in 3,4,5-THB and 2,5-DHB; these acids showed the highest antioxidant potential in the DPPH, CUPRAC, and FRAP tests.

In the case of the proton of the carboxyl group, higher values of chemical shifts were observed in the ^1^H NMR spectra of 3,4 and 3,5-dihydroxybenzoic acids than in the case of other acids. 

### 3.14. Statistical Analysis 

**Figure 9:**
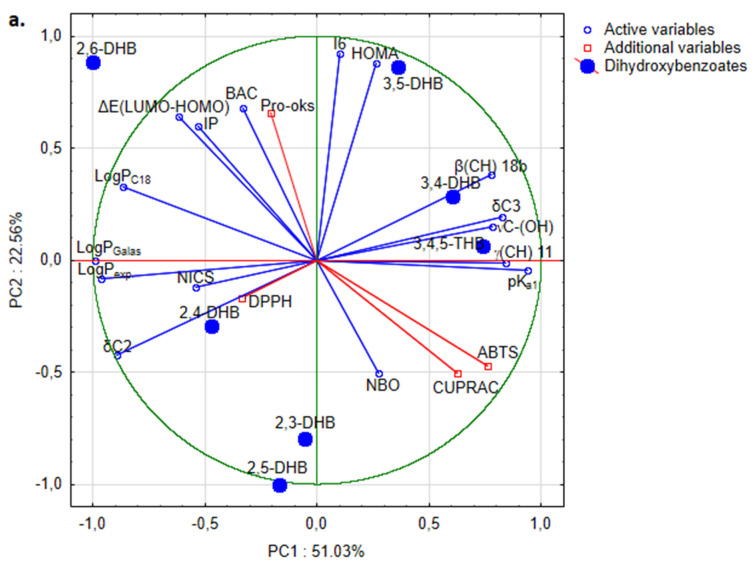

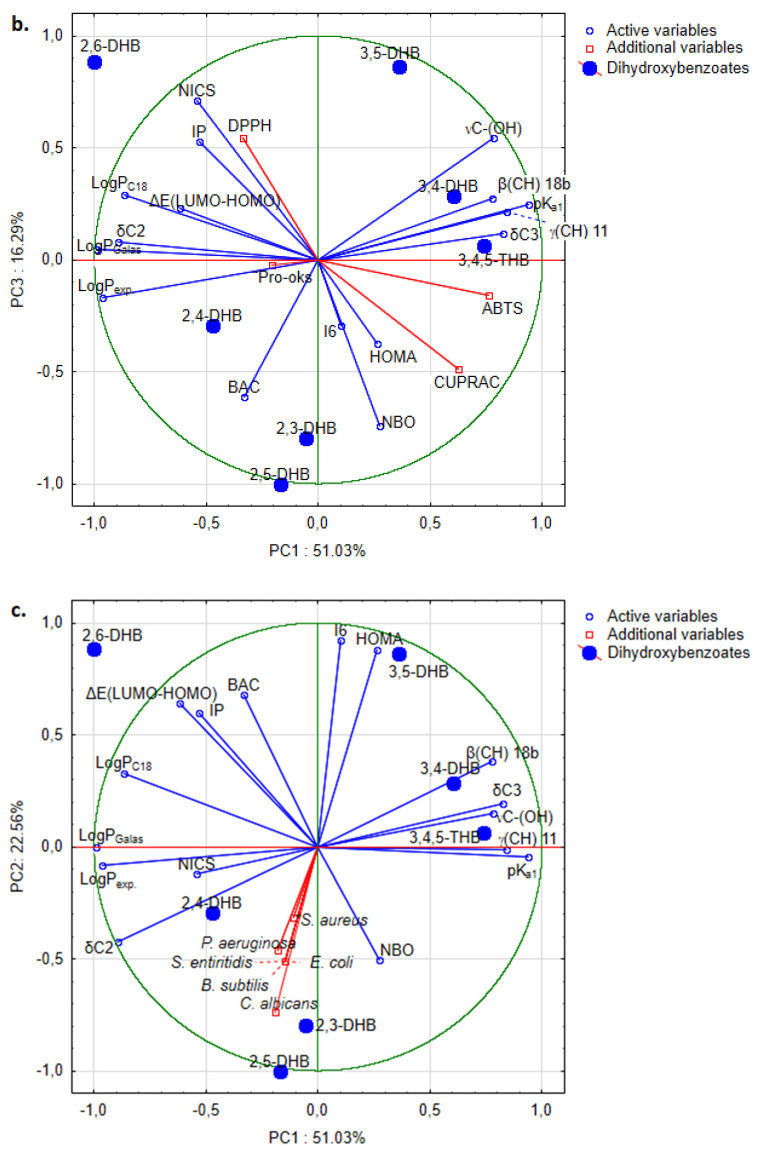

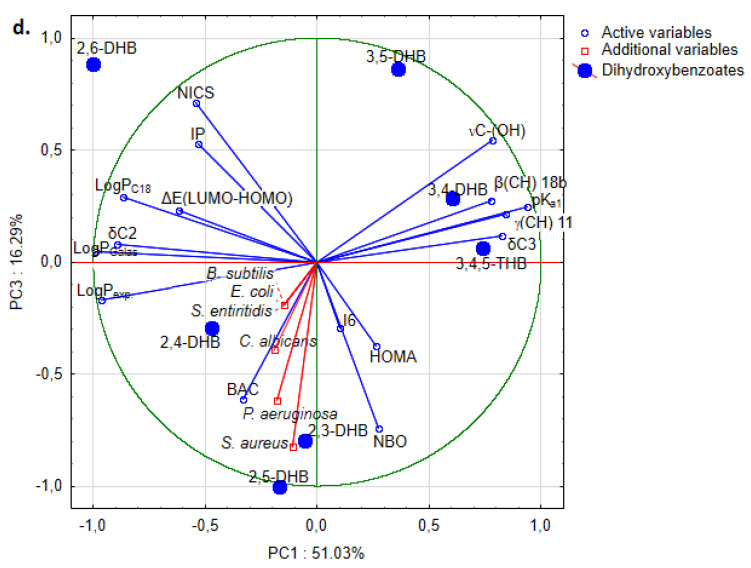
Principal component analysis (PCA) of lipophilic and electronic parameters, aromaticity indices, FTIR and NMR parameters, and the anti-/pro-oxidant (**a**,**b**) and antimicrobial potential (**c**,**d**) of the studied compounds.

The relationships between the lipophilic and electronic parameters, aromaticity indices, FTIR and NMR parameters, and anti-/pro-oxidant and antimicrobial potential of the selected dihydroxybenzoates were evaluated by principal component analysis. The list of variables correlated with one another is summarized in Table S7. Predominant highly significant correlation coefficients between variables proved the application of PCA to be justified and promising; however, lipophilic and electronic parameters, aromaticity indices, and FTIR and NMR parameters (those that significantly change position in the spectra depending on the number and location of the substituents in the ring) were used as input variables to the PCA, while anti-/pro-oxidant and antimicrobial potential were used as accompanying variables. According to the PCA and the Kaiser criteria, three principal components with eigenvalues > 1 were obtained, accounting for 89.9% of the total variance. PC1, with an eigenvalue equal to 8.16 explaining 51.0% of the total variance, was contributed to by logP_Galas_, logP_exp_., δC2, logP_C18_, and ΔE_(LUMO-HOMO)_ with negative correlation, and by pK_a1_, γ(CH) 11, δC3, νC-(OH), and β(CH) 18b with positive correlation (Table S8). Based on the characteristics contributing to the first principal component, this reflects the hydrophilic–hydrophobic and spectral characteristics of dihydroxybenzoates. PC2, with an eigenvalue equal 3.61, explains around 22.6% of the total variance, and shows directly proportional correlation between I6, HOMA, BAC, IP, and ΔE_(LUMO-HOMO)_. This could be accepted as a component indicating some aromaticity due to the energy difference between the frontier orbitals. PC3, with an eigenvalue 2.61 explaining 16.3% of the total variance, was contributed to by positive correlation between NICS, νC-(OH), and IP, as well as negative correlation between BAC and NBO, mainly reflecting the atomic charge and electric characteristics. Biplots of factor loadings and sample scores of all combination of PCs with corresponding anti-/pro-oxidant and antimicrobial potential are presented on Figure 9a–d, respectively. The antioxidant activity of the studied compounds described by the CUPRAC and ABTS parameters correlates with PC1, DPPH parameters (IC_50_) correlate with PC3, whereas pro-oxidant activity correlates with PC2. The antioxidant parameters obtained via the ABTS and CUPRAC assays negatively correlate with the DPPH assay’s parameter. Compounds with high scavenging activity against DPPH^•^ (lower IC_50_ values) possess high antioxidant activity as measured by the ABTS and CUPRAC assays. Moreover, the pro-oxidant activity of HBAs is negatively correlated with antioxidant properties (compounds with pro-oxidant activity—2,6-, 3,5-, and 3,4-DHB—are weaker antioxidants compared with the other HBAs). The antimicrobial properties of HBAs correlate with PC2 and PC3. 2,3- and 2,5-DHB possess the lowest antimicrobial activity (compared with other HBAs, as expressed by their generally having the highest MIC values), and are characterized by lower IP, ΔE_(LUMO-HOMO)_, and BAC values than the other HBAs. PCA analysis showed differences between the HBAs that can be divided into groups taking into account the parameters used for calculations of the PCs: (a) 3,4,5-THB, 3,4-DHB, and 3,5-DHB (higher values of spectral parameters, lower values of logP, higher pK_a_), (b) 2,5-DHB, 2,3-DHB, and 2,4-DHB. 

## 4. Conclusions

The number and position of hydroxyl substituents in the benzoic acid moiety strongly affect the electronic charge distribution over the molecules, as well as their biological properties. PCA analysis was successfully applied to describe the structure–activity relationship in the group of HBAs. Three principal components described 89.9% of the total variance. The applied variables describing the structure of HBAs included lipophilic and electronic parameters, aromaticity indices, NBO charges, and spectral (FTIR and NMR) parameters. The antioxidant activity—described by parameters obtained via the DPPH, ABTS, FRAP, and CUPRAC assays—strongly correlates with the calculated electronic parameters (Table S2) and NBO and CHelpG charges. With the decrease in the total electronic charge of the aromatic ring and the increase in electronic charge gathered on the -OH group, the electron-/hydrogen-donating properties of molecules also increase. Because the antioxidant assays were based on different mechanisms and factors, such as type of solvent, pH may affect the results. The PCA analysis showed that the CUPRAC and ABTS parameters correlate with PC1, the DPPH parameter (IC_50_) correlates with PC3, and pro-oxidant activity correlates with PC2. This suggests that different structural parameters can play a significant role in assessing antioxidant activity, depending on the test used. Therefore, it is not possible to rank the HBAs according to antioxidant activity, but taking into account the obtained parameters it can be stated that 3,4,5-THB and 3,4-, 2,3-, and 2,5-DHB showed the highest antioxidant activity. The position of the hydroxyl groups in the *meta-* position in relation to one another reduces the antioxidant and antiradical properties of hydroxybenzoic acids. 2,4-, 3,4-, and 3,5-DHB showed the highest antimicrobial activity against *E. coli*, *P. aeruginosa*, *S. aureus*, *B. subtilis*, *S. enteritidis*, and *C. albicans*. These compounds are characterized by low-to-moderate lipophilicity (logP_exp._ in the range 0.86–1.63) and acidity at the level of pK_a_ 3.11–4.26. Two of them—3,4- and 3,5-DHBs—showed pro-oxidant activity in the Trolox oxidation test, but were non-cytotoxic to the MCF-7 and MDA-MB-231 cell lines. The highest cytotoxicity was observed in 3,4,5-THB (IC_50_ = 0.44 ± 1.80 and IC_50_ = 0.36 ± 0.50 respectively for MCF-7, and MDA-MB-231), which also possessed the highest antioxidant activity in the DPPH and CUPRAC assays, and no pro-oxidant activity in the Trolox oxidation test. 3,4,5-THB had the lowest lipophilicity and acidity among the studied compounds. Taking into account these two parameters, the HBAs can be divided into three groups that are characterized by: (a) lower acidity and lipophilicity (3,4,5-THB; 3,4-DHB; 3,5-DHB), (b) moderate acidity and lipophilicity (2,3-DHB; 2,4-DHB; 2,5-DHB), and (c) higher acidity and lipophilicity (2,6-DHB).

The HBAs commonly present in plant-based foods have significant biological properties. Some of them can be applied as strong antioxidants, antimicrobials, or even as chemopreventive or anticancer agents. Their mechanism of action is still not elucidated, and requires further study. It is worth undertaking research on the mechanism of action of HBAs at the cellular level, because some of them have the potential to be used as dietary supplements, functional food ingredients, or in the prevention and treatment of diseases. 

## Figures and Tables

**Table 1:** DPPH antioxidant activity of the tested hydroxybenzoic acids (methanolic solutions) measured after 60 min of reaction time. Mean values from three independent experiments ± SD are shown.

Compound	2,3-DHB	2,4-DHB	2,5-DHB	2,6-DHB	3,4-DHB	3,5-DHB	3,4,5-THB
DPPH/IC_50_ (µM)	5.86 ± 0.64	>120,000	4.99 ± 0.79	209.02 ± 0.98	5.24 ± 0.95	>1000	2.42 ± 0.08

**Table 2:** Antioxidant activity of selected hydroxybenzoic acids on the basis of literature data.

Method	2,3-DHB	2,4-DHB	2,5-DHB	2,6-DHB	3,4-DHB	3,5-DHB	3,4,5-THB
DPPH, IC_50_ (μM)	3.52 * (C_DPPH_ = 1 mM, t = 30 min) [63]		4.95 (C_DPPH_ = 60 μM, t = 60 min) [62]	231.51 (C_DPPH_ = 500 μM, t = 30 min) [64]	3.91 * (C_DPPH_ = 1 mM, t = 30 min) [63]	384.6 (C_DPPH_ = 90 μM, t = 30 min) [65]	3.78 * (C_DPPH_ = 1 mM, t = 30 min) [63]
			5.18 * (C_DPPH_ = 1 mM, t = 30 min) [63]	15.38 * (C_DPPH_ = 1 mM, t = 30 min) [63]	3.83 (C_DPPH_ = 50 μM, t = 30 min) [66]		0.0237 (C_DPPH_ = 1 mM, t = 20 min) [67]
			37.04 (C_DPPH_ = 90 μM, t = 30 min) [65]		0.0574 (C_DPPH_ = 1 mM, t = 20 min) [67]		
			0.0292 (C_DPPH_ = 1 mM, t = 20 min) [67]		15.83 (C_DPPH_ = 60 μM, t = 30 min) [68]		
			6.30 (C_DPPH_ = 60 μM, t = 30 min) [68]				
DPPH radical scavenging activity (%)	46 (C_2,3-DHB_ = 0.11 mg/mL, C_DPPH_ = 100 μM, t = 1 min) [69]	0.11 (C_2,4-DHB_ = 0.11 mg/mL, C_DPPH_ = 100 μM, t = 1 min) [69]	30.5 (C_2,5-DHB_ = 0.11 mg/mL, C_DPPH_ = 100 μM, t = 1 min) [69]		41.2 (C_3,4-DHB_ = 0.11 mg/mL, C_DPPH_ = 100 μM, t = 1 min) [69]	0.6 (C_3,5-DHB_ = 0.11 mg/mL, C_DPPH_ = 100 μM, t = 1 min) [69]	75 (C_3,4,5-THB_ = 0.11 mg/mL, C_DPPH_ = 100 μM, t = 1 min) [69]
					≈ 70 (C_3,4-DHB_ = 10 μM, C_DPPH_ = 100 μM, t = 30 min) [70]		≈ 70 (C_3,4,5-THB_ = 10 μM, C_DPPH_ = 100 μM, t = 30 min) [70]
					≈ 88.5 (C_3,4-DHB_ = 15 μM, C_DPPH_ = 60 μM, t = 30 min) [71]		40 (C_3,4,5-THB_≈ 0.7 mM, C_DPPH_ = 200 μM, t = 30 min) [72]
							88.5 (C_3,4,5-THB_ = 15 μM, C_DPPH_ = 60 μM, t = 30 min) [71]
ABTS, IC_50_ (μM)	10.73 (C_ABTS_ = 7 mM, t = 6 min) [73]	169.44 (C_ABTS_ = 7 mM, t = 6 min) [73]	15.56 (C_ABTS_ = 7 mM, t = 6 min) [73]		20.67 (C_ABTS_ = 7 mM, t = 6 min) [73]	41.85 (C_ABTS_ = 7 mM, t = 6 min) [73]	4.33 (C_ABTS_ = 7 mM, t = 6 min) [73]
			≈ 6.50 (C_ABTS_ = 7 mM, t = 6 min) [68]		0.89 * (C_ABTS_ = n.d., t = 6 min) [74]		
					10.07 (C_ABTS_ = 7 mM, t = 6 min) [68]		
ABTS radical scavenging activity (%)					≈ 38 (C_3,4,5-THB_ = 10 μM, C_ABTS_ = 7 mM, t = 7 min) [71]		≈ 80 (C_3,4,5-THB_ = 10 μM, C_ABTS_ = 7 mM, t = 7 min) [71]
FRAP (μM Fe^2+^)		≈ 0.012 (C = 0,001 mg/mL), λ = 593 nm, t = 30 min) [73]	≈ 0.98 (C = 0,001 mg/mL), λ = 593 nm, t = 30 min) [73]		≈ 0.65 (C = 0,001 mg/mL), λ = 593 nm, t = 30 min) [73]	≈ 0.03 (C = 0,001 mg/mL), λ = 593 nm, t = 30 min) [73]	≈ 1.03 (C = 0,001 mg/mL), λ = 593 nm, t = 30 min) [73]
			75.39 (C = 5 μM, λ = 594 nm, t = n.d.) [62]				
			236.86 (C = 50 μM, λ = 594 nm, t = n.d.) [62]				

* Results expressed as μg/L; ≈ the exact value was not given; n.d.: no data.

**Table 3:** FRAP antioxidant activity of tested hydroxybenzoic acids at a concentration of 50 μM (methanolic solutions). Mean values from three independent experiments ± SD are shown.

Compound	2,3-DHB	2,4-DHB	2,5-DHB	2,6-DHB	3,4-DHB	3,5-DHB	3,4,5-THB
FRAP (µM Fe^2+^)	173.79 ± 39.06	(-) *	236.00 ± 6.31	(-) *	44.22 ± 9.38	(-) *	158.10 ± 14.81

* Negative values were obtained for these acids.

**Table 4:** The minimum inhibitory concentration (MIC) values (mg/mL) for the tested hydroxybenzoic acids against selected microorganisms.

Microorganisms	Compound						
	2,3-DHB	2,4-DHB	2,5-DHB	2,6-DHB	3,4-DHB	3,5-DHB	3,4,5-THB
	MIC (mg/mL)						
*Escherichia coli*	5	2	3	3	2	2	3
*Pseudomonas aeruginosa*	5	2	5	4	2	3	4
*Staphylococcus aureus*	5	2	5	5	2	2	6
*Bacillus subtilis*	5	2	3	3	2	2	3
*Salmonella enteritidis*	5	2	3	3	2	2	3
*Candida albicans*	5	2	5	3	2	2	3

**Table 5:** IC_50_ values for the tested hydroxybenzoic acids on breast cancer cells. Mean values from three independent experiments ± SD are shown.

Compound	Cytotoxicity (mM) in Cell Line	
	MDA-MB-231	MCF-7
2,3-DHB	IC_50_ = 4.10 ± 0.76	NT *
2,4-DHB	IC_50_ = 4.77 ± 1.20	NT *
2,5-DHB	IC_50_ = 4.39 ± 1.50	NT *
2,6-DHB	IC_50_ = 3.97 ± 2.50	NT *
3,4-DHB	NT *	NT *
3,5-DHB	NT *	NT *
3,4,5-THB	IC_50_ = 0.36 ± 0.50	IC_50_ = 0.44 ± 1.80

* NT: nontoxic.

**Table 6:** Lipophilicity parameters determined by chromatographic methods (the logarithm of the retention factor, log_kw_) and octanol/water partition coefficients logP obtained for hydroxybenzoic acids.

Compound	C18			C8			CN			IAM			PHE		
	log k_w_	ϕ_0_	R^2^	log k_w_	ϕ_0_	R^2^	log k_w_	ϕ_0_	R^2^	log k_w_	ϕ_0_	R^2^	log k_w_	ϕ_0_	R^2^
2,3-DHB	2.47	0.60	0.950	1.70	0.87	0.994	1.59	1.16	0.997	2.17	1.08	0.996	1.25	0.93	0.972
2,4-DHB	2.66	0.57	0.983	1.81	1.38	0.981	2.06	1.15	0.999	1.14	1.00	0.995	1.39	1.05	0.996
2,5-DHB	2.39	0.55	0.985	1.37	0.90	0.993	1.64	1.15	0.997	1.60	1.11	0.997	1.17	1.01	0.986
2,6-DHB	2.99	0.68	0.926	1.17	1.05	0.927	1.49	1.08	0.975	1.31	1.14	0.989	0.87	1.05	0.998
3,4-DHB	2.32	0.45	0.979	1.00	0.83	0.988	1.58	1.08	0.996	0.44	1.13	0.993	1.07	0.50	0.996
3,5-DHB	2.56	0.45	0.983	0.90	0.83	0.978	1.78	1.07	0.994	0.51	1.16	0.993	1.03	0.55	0.985
3,4,5-THB	1.91	0.37	0.977	0.50	0.91	0.979	1.24	1.06	0.977	0.15	1.44	0.989	0.83	0.33	0.961

**Table 7:** Theoretical (calculated in ACD/Labs or Percepta: logP classic, logP galas, pKa classic) and experimental logP and pKa values.

Compound	LogP Classic	LogP Galas	LogP_exp._	ACD/pK_a_ Classic	pKa_exp._
2,3-DHB	1.80 ± 0.26	1.42	1.20 [102]	pK_a1_ = 3.0 ± 0.1 pK_a2_ = 10.1 ± 0.1 pK_a3_ = 15.6 ± 0.1	2.91 (at 25 °C) [103] 2.98 [104] 2.56 [105]
2,4-DHB	1.60 ± 0.26	1.82	1.63 [102]	pK_a1_ = 3.3 ± 0.1 pK_a2_ = 9.1 ± 0.2 pK_a3_ = 13.8 ± 0.1	3.11 (at 25 °C) [106] 3.11 [104] 3.1 [105]
2,5-DHB	1.56 ± 0.26	1.55	1.74 [107]	pK_a1_ = 3.0 ± 0.1 pK_a2_ = 11.0 ± 0.2 pK_a3_ = 14.7 ± 0.2	2.97 (at 25 °C) [104] 2.84 [105] 2.97 [108]
2,6-DHB	2.24 ± 0.27	2.32	2.20 [102]	pK_a1_ = 1.3 ± 0.1 pK_a2_ = 12.5 ± 0.1 pK_a3_ = 14.1 ± 0.1	1.30 [104] 1.51 [105]
3,4-DHB	1.16 ± 0.24	0.97	0.86 [102]	pK_a1_ = 4.5 ± 0.1 pK_a2_ = 9.1 ± 0.2 pK_a3_ = 12.8 ± 0.1	4.26 (at 25 °C) [109] 4.48 [104] 4.16 [105]
3,5-DHB	1.12 ± 0.24	0.99	0.86 [102]	pK_a1_ = 4.0 ± 0.1 pK_a2_ = 9.8 ± 0.1 pK_a3_ = 11.3 ± 0.1	4.04 [104] 3.61 [105] 4.04 [108]
3,4,5-THB	0.91 ± 0.33	0.61	0.70 [102]	pK_a1_ = 4.3 ± 0.1 pK_a2_ = 9.0 ± 0.2 pK_a3_ = 12.3 ± 0.1 pK_a4_ = 14.4 ± 0.2	4.40 [110] 4.41 [104] 3.94 [105]

## Data Availability

The data presented in this study are available on request from the corresponding author.

## Supplementary Materials

The following are available online at https://www.mdpi.com/article/10.3390/nu13093107/s1: Figure S1: The FTIR spectra of hydroxybenzoic acids; Figure S2: ^1^H and ^13^C NMR spectra of hydroxybenzoic acids; Figure S3. The atom numbering scheme; Table S1: Pearson’s correlation matrix (*p* < 0.05) of different theoretical and experimental logP and pK_a_ parameters; Table S2: Calculated parameters of chemical reactivity of tested hydroxybenzoic acids in gas, aqueous, and methanolic phases; Table S3: The values of aromaticity indexes of the tested acids (Aj, BAC, HOMA, GEO, EN, I6, NICS) calculated for structures optimized in the gas and aqueous phases using the B3LYP/6-311++G(d,p) method; Table S4: The distribution of electronic charges (calculated via ChelpG and NBO) for dihydroxybenzoic acids and trihydroxybenzoic (gallic) acid; Table S5: Wavenumbers (cm^−1^), intensities, and assignments of bands occurring in the experimental FTIR spectra of hydroxybenzoic acids; Table S6: Chemical shifts δ (ppm) in the ^1^H and ^13^C NMR spectra of hydroxybenzoic acids; Table S7: Pearson’s correlation coefficient matrix between lipophilic and electronic parameters, aromaticity indices, and FTIR and NMR parameters of the selected dihydroxybenzoates; Table S8: PCA factor loadings, eigenvalues, and explained percentage of variance.
