# Supplementary material for: Plant-Derived and Dietary Hydroxybenzoic Acids—A Comprehensive Study of Structural, Anti-/Pro-Oxidant, Lipophilic, Antimicrobial, and Cytotoxic Activity in MDA-MB-231 and MCF-7 Cell Lines
Source: Nutrients. 2021 Sep 4;13(9):3107. doi: 10.3390/nu13093107 (PMC8466373; doi:10.3390/nu13093107)
Supplement: Supplementary file 1 [file nutrients-13-03107-s001.zip › nutrients-1353070-supplementary.pdf]

Supplementary materials

Plant derived and dietary hydroxybenzoic acids – a comprehensive study of structural, anti-/pro-oxidant, lipophilic, antimicrobial and cytotoxic activity in MDA-MB-231 and MCF-7 cell lines

Monika Kalinowska[1,*], Ewelina Gołębiewska[1], Grzegorz Świderski[1], Sylwia Męczyńska-Wielgosz[2], Hanna Lewandowska[3], Anna Pietryczuk[4], Adam Cudowski[4], Aleksander Astel[5], Renata Świsłocka[1], Mariola Samsonowicz[1], Anna Barbara Złowodzka[6], Waldemar Priebe[7] and Włodzimierz Lewandowski[8]

[1] Department of Chemistry, Biology and Biotechnology, Institute of Civil Engineering and Energetics, Faculty of Civil Engineering and Environmental Science, Bialystok University of Technology, Wiejska 45E Street, 15-351 Bialystok, Poland; m.kalinowska@pb.edu.pl (M.K.); e.golebiewska@pb.edu.pl (E.G.); g.swiderski@pb.edu.pl (G.Ś.); r.swislocka@pb.edu.pl (R.Ś.); m.samsonowicz@pb.edu.pl (M.S.); bzlowodzka@gmail.com (A.B.Z); w-lewando@wp.pl (W.L.)

[2] Centre of Radiobiology and Biological Dosimetry, Institute of Nuclear Chemistry and Technology, Dorodna 16 Street, 03-195 Warsaw, Poland; s.meczynska@ichtj.waw.pl (S.M.W.)

[3] Institute of Nuclear Chemistry and Technology, 16 Dorodna street, 03-195, Warsaw, Poland; h.lewandowska@ichtj.waw.pl (H.L.)

[4] Department of Water Ecology, Faculty of Biology, University of Bialystok, Ciolkowskiego 1J Street, 15-245 Bialystok, Poland; annapiet@uwb.edu.pl (A.P.); cudad@uwb.edu.pl (A.C.)

[5] Environmental Chemistry Research Unit, Institute of Biology and Earth Sciences, Pomeranian University in Słupsk, Arciszewskiego 22a Street, 76-200 Słupsk, Poland; aleksander.astel@apsl.edu.pl (A.A.)

6 Faculty of Chemistry, Warsaw University of Technology, Noakowskiego 3 Street, 00-664 Warszawa; bzlowodzka@gmail.com (A.B.Z.)

[7] Department of Experimental Therapeutics, The University of Texas MD Anderson Cancer Center, 1901 East Rd., Houston, TX 77054, USA; wpriebe@mac.com (W.P.)

[8] Institute of Agricultural and Food Biotechnology—State Research Institute, Warsaw, Poland; w-lewando@wp.pl (W.L.)

*Correspondence: m.kalinowska@pb.edu.pl (M.K.)

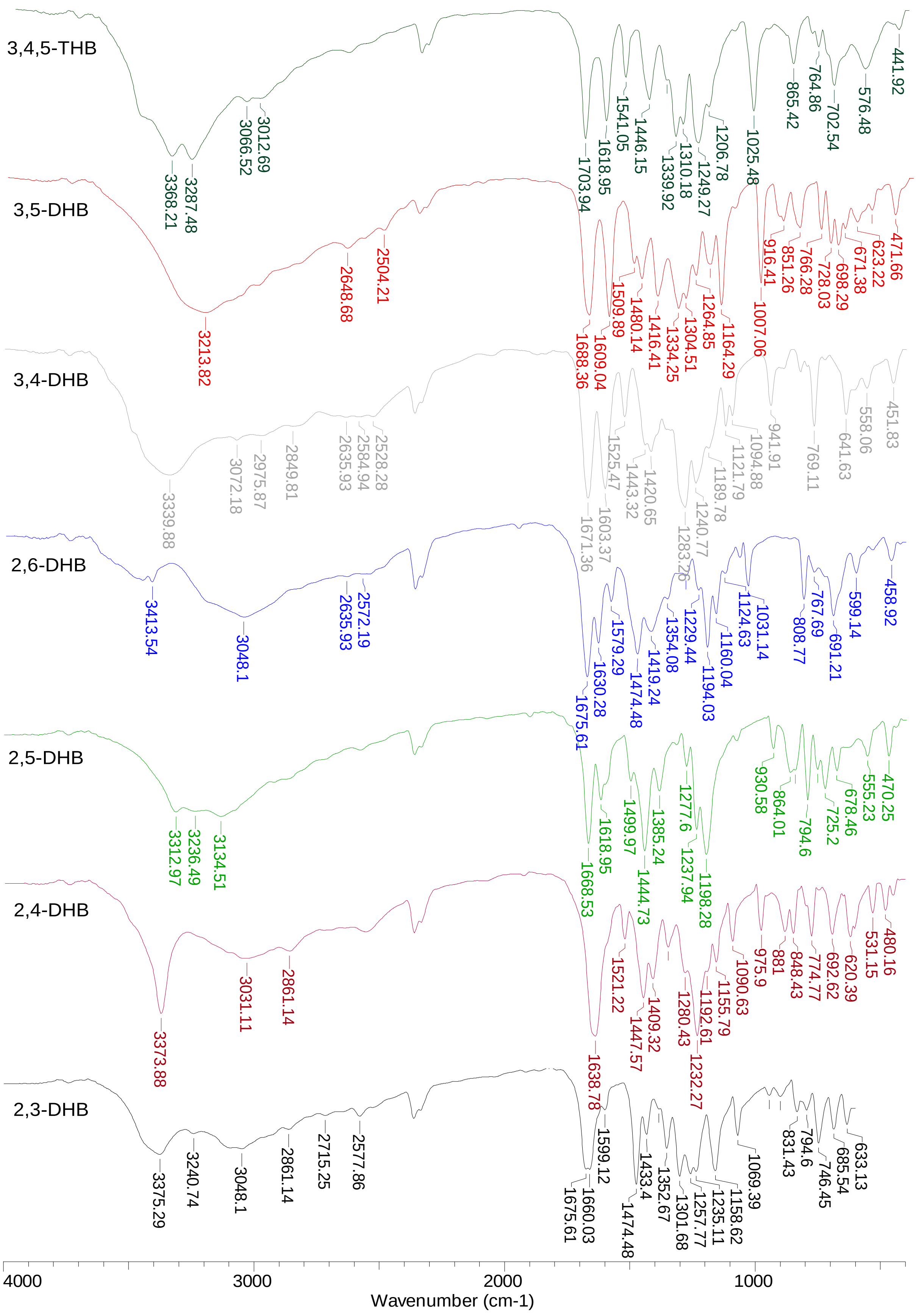

Figure S1. The FT-IR spectra of hydroxybenzoic acids.

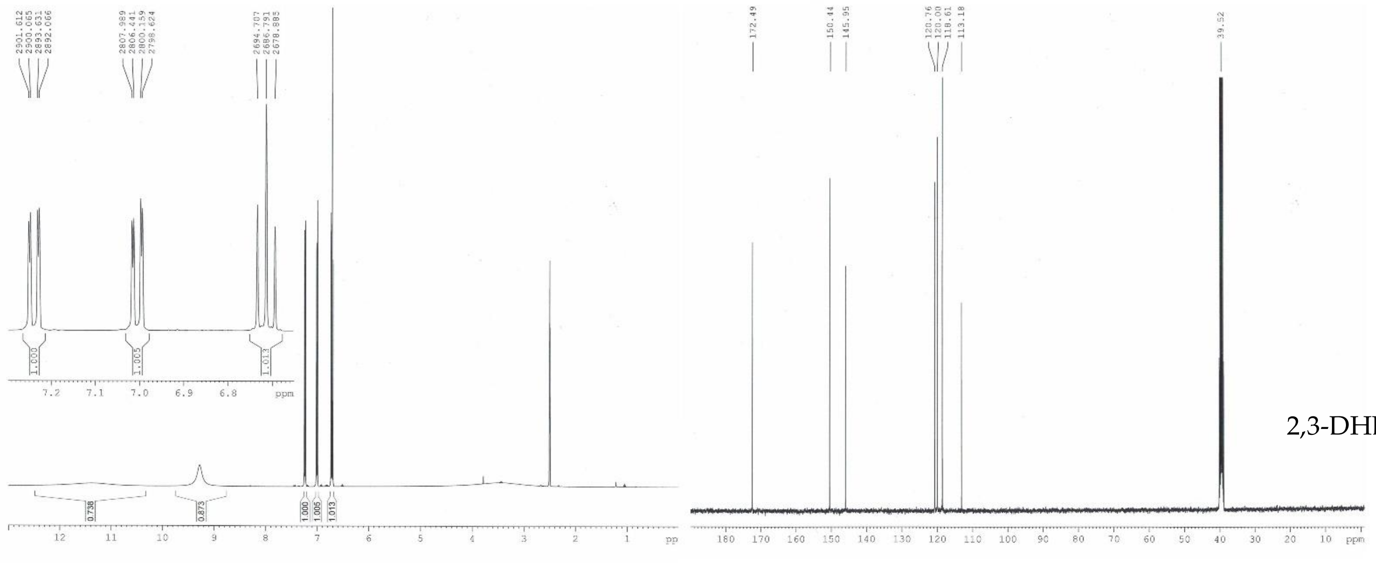
172.49
150.44
145.95
120.76
120.00
118.61
113.18
39.52
ppm

2,3-DHB

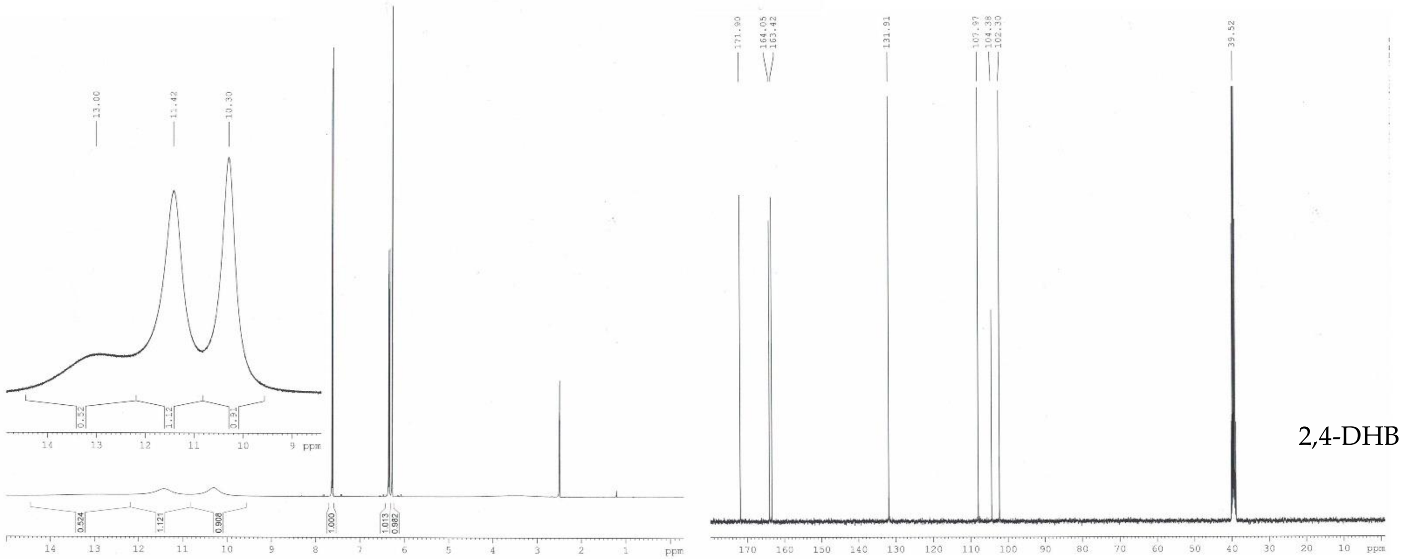
13.00
11.42
10.30
171.90
164.05
163.42
131.91
107.97
104.38
102.30
39.52
ppm

2,4-DHB

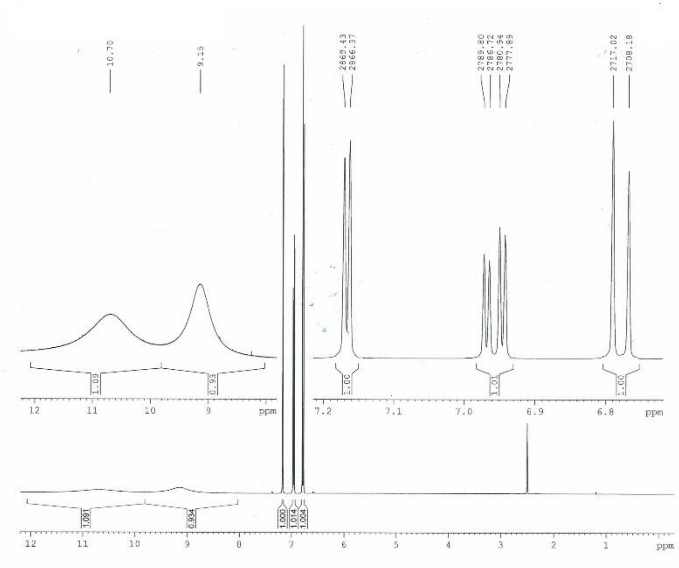

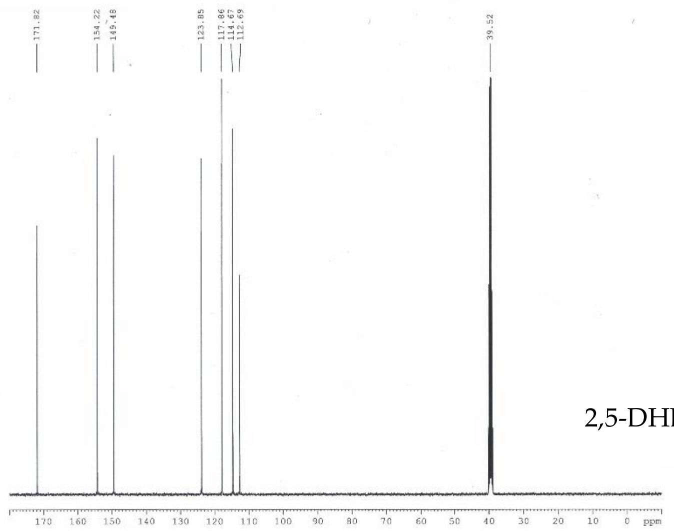

2,5-DHB

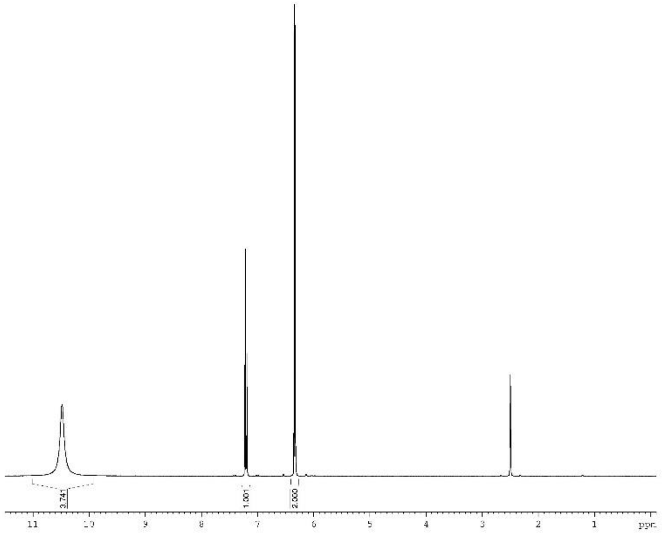

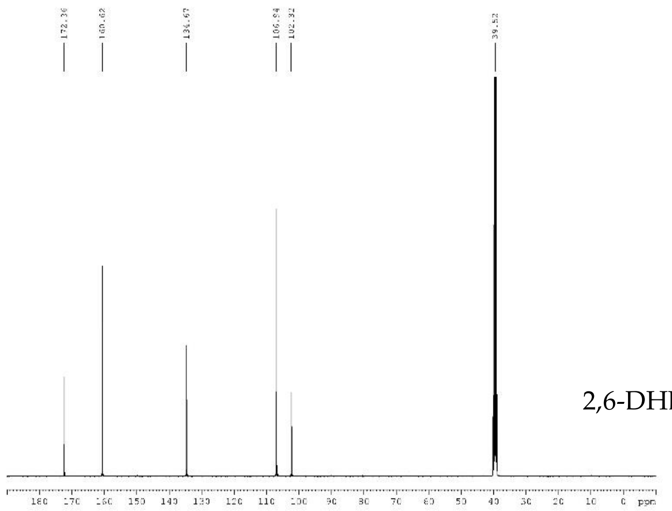

2,6-DHB

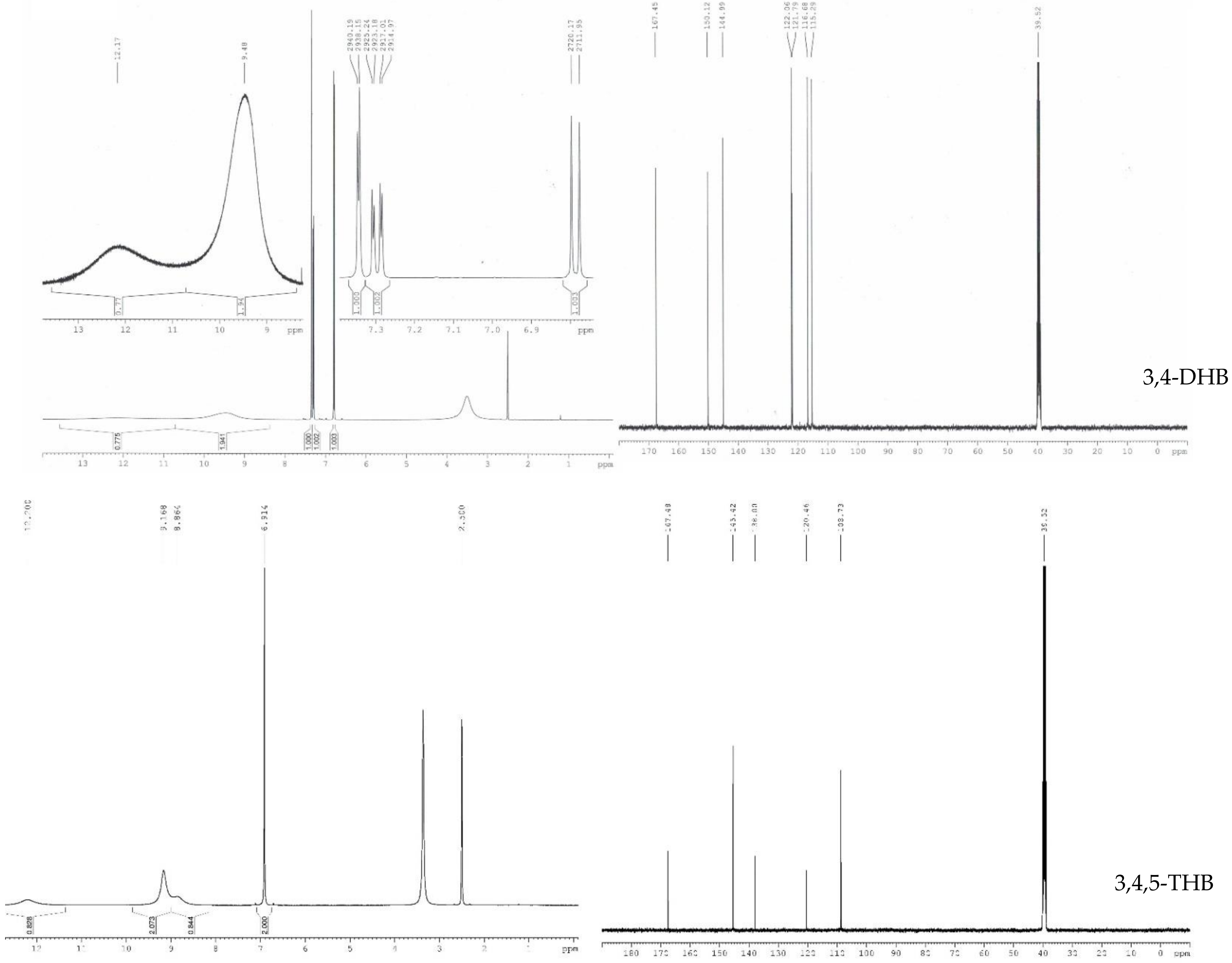

Figure S2. ^{1}H and ^{13}C NMR spectra of hydroxybenzoic acids. Data for 3,5-DHB come from the literature [114].

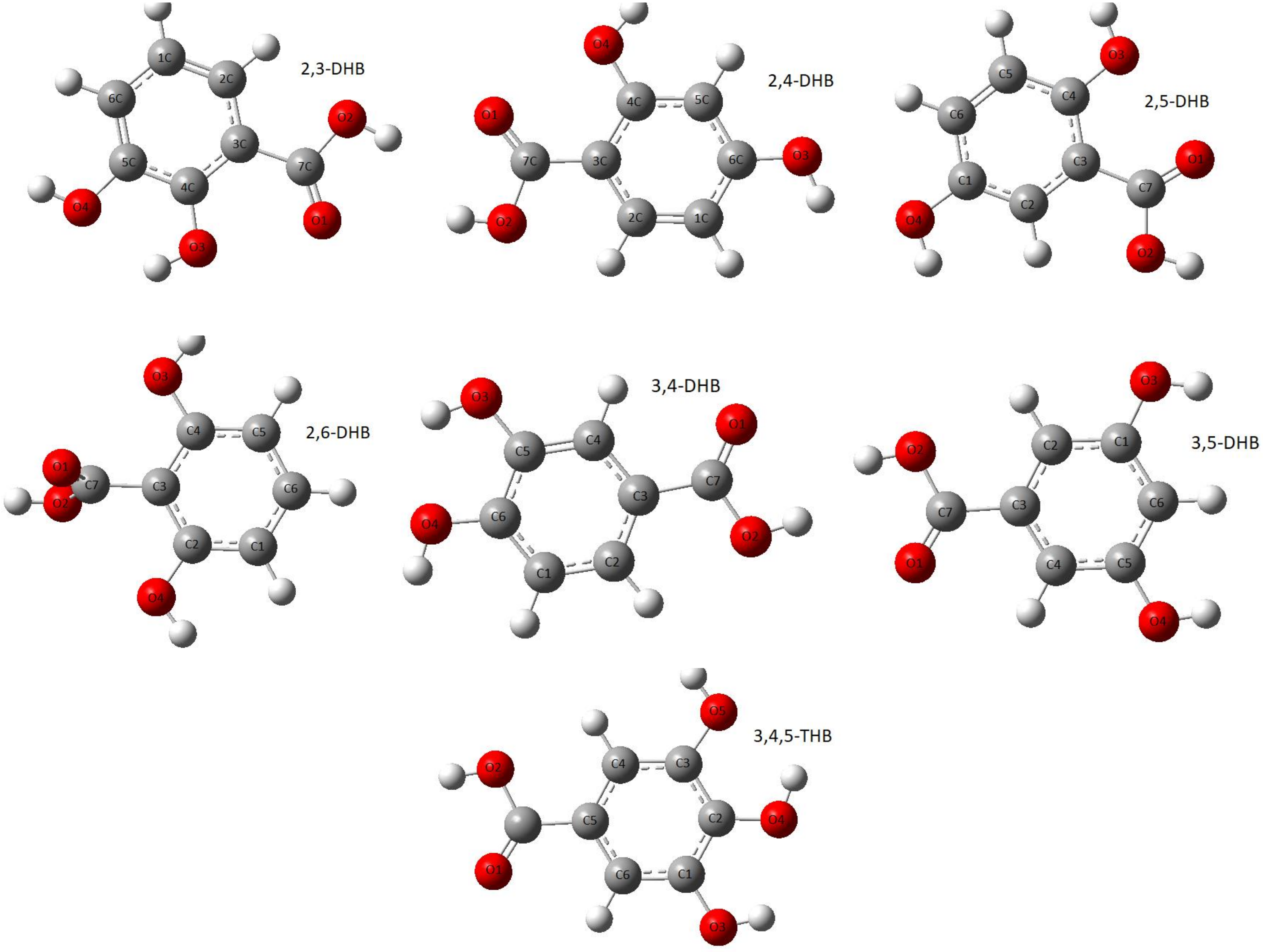

Figure S3. The atom numbering scheme.

Table S1. Person's correlation matrix ($p<0.05$) of different theoretical and experimental logP and pK_a parameters.

	**LogP $_{C18}$**	**LogP$_{C8}$**	**LogP$_{CN}$**	**LogP$_{IAM}$**	**LogP$_{PHE}$**	**LogP$_{Classic}$**	**LogP$_{Galas}$**	**pK_{a1}**	**pK_{a2}**	**pK_{a3}**	**LogP$_{exp}$**
**LogP $_{C18}$**	1.000										
**LogP$_{C8}$**	0.527	1.000									
**LogP$_{CN}$**	0.493	0.697	1.000								
**LogP$_{IAM}$**	0.459	0.813	0.241	1.000							
**LogP$_{PHE}$**	0.199	0.879	0.813	0.544	1.000						
**LogP$_{Classic}$**	0.821	0.629	0.169	0.766	0.186	1.000					
**LogP$_{Galas}$**	0.882	0.641	0.364	0.629	0.253	0.943	1.000				
**pK_{a1}**	-0.784	-0.430	-0.033	-0.645	0.033	-0.953	-0.919	1.000			
**pK_{a2}**	0.677	0.150	-0.166	0.504	-0.280	0.804	0.749	-0.905	1.000		
**pK_{a3}**	0.291	0.750	0.065	0.925	0.477	0.730	0.594	-0.600	0.432	1.000	
**LogP$_{exp}$**	0.774	0.544	0.254	0.598	0.166	0.892	0.965	-0.918	0.806	0.597	1.000

Table S2. Calculated parameters of chemical reactivity of tested hydroxybenzoic acids in gas, aqueous and methanolic phases.

Parameters [eV]	Compound						
	**2,3-DHB**	**2,4-DHB**	**2,5-DHB**	**2,6-DHB**	**3,4-DHB**	**3,5-DHB**	**3,4,5-THB**
				**Gas phase**			
E_{HOMO}	-8.9204	-9.2813	-8.7787	-9.2462	-8.9468	-9.2467	-8.7735
E_{LUMO}	-5.4877	-5.3359	-5.7051	-4.9974	-5.3824	-5.6616	-5.3677
$\Delta E_{(LUMO-HOMO)}$	3.4327	3.9454	3.0735	4.2488	3.5644	3.5851	3.4058
Ionization potential (IP)	8.9204	9.2813	8.7787	9.2462	8.9468	9.2467	8.7735
Electron affinity (A)	5.4877	5.3359	5.7051	4.9974	5.3824	5.6616	5.3677
Electronegativity (χ)	7.2041	7.3086	7.2419	7.1218	7.1646	7.4542	7.0706
Electronic chemical potential (μ)	-7.2041	-7.3086	-7.2419	-7.1218	-7.1646	-7.4542	-7.0706
Chemical hardness (η)	1.7164	1.9727	1.5368	2.1244	1.7822	1.7925	1.7029
Chemical softness (σ)	0.2913	0.2535	0.3254	0.2354	0.2806	0.2789	0.2936
Electrophilicity index (ω)	15.1189	13.5387	17.0365	11.9374	14.4012	15.4987	14.6790
				**Aqueous solution**			
E_{HOMO}	-8.9153	-9.2753	-8.7749	-9.2475	-8.9340	-9.2290	-8.7555
E_{LUMO}	-5.5016	-5.3484	-5.7128	-5.0929	-5.3919	-5.6660	-5.3721
$\Delta E_{(LUMO-HOMO)}$	3.4137	3.9269	3.0621	4.1546	3.5421	3.5631	3.3835
Ionization potential (IP)	8.9153	9.2753	8.7749	9.2475	8.9340	9.2290	8.7555
Electron affinity (A)	5.5016	5.3484	5.7128	5.0929	5.3919	5.6660	5.3721
Electronegativity (χ)	7.2084	7.3118	7.2438	7.1702	7.1630	7.4475	7.0638
Electronic chemical potential (μ)	-7.2084	-7.3118	-7.2438	-7.1702	-7.1630	-7.4475	-7.0638
Chemical hardness (η)	1.7068	1.9634	1.5310	2.0773	1.7711	1.7815	1.6917
Chemical softness (σ)	0.2929	0.2547	0.3266	0.2407	0.2823	0.2807	0.2956
Electrophilicity index (ω)	15.2216	13.6146	17.1362	12.3746	14.4853	15.5667	14.7474
				**Methanolic solution**			
E_{HOMO}	-8.9155	-9.2755	-8.7751	-9.2475	-8.9343	-9.2296	-8.7550
E_{LUMO}	-5.5011	-5.3479	-5.7125	-5.0893	-5.3917	-5.6660	-5.3723
$\Delta E_{(LUMO-HOMO)}$	3.4145	3.9277	3.0626	4.1582	3.5427	3.5636	3.3826
Ionization potential (IP)	8.9155	9.2755	8.7751	9.2475	8.9343	9.2296	8.7550
Electron affinity (A)	5.5011	5.3479	5.7125	5.0893	5.3917	5.6660	5.3723
Electronegativity (χ)	7.2083	7.3117	7.2438	7.1684	7.1630	7.4478	7.0637
Electronic chemical potential (μ)	-7.2083	-7.3117	-7.2438	-7.1684	-7.1630	-7.4478	-7.0637
Chemical hardness (η)	1.7072	1.9638	1.5313	2.0791	1.7713	1.7818	1.6913
Chemical softness (σ)	0.2929	0.2546	0.3265	0.2405	0.2832	0.2806	0.2956
Electrophilicity index (ω)	15.2174	13.6113	17.1332	12.3579	14.4831	15.5654	14.7504

Table S3. The values of aromaticity indexes of the tested acids (Aj, BAC, HOMA, GEO, EN, I6, NICS) calculated for structures optimized in the gas and water phase using the B3LYP / 6-311 ++ G (d,p) method.

	2,3-DHB	2,4-DHB	2,5-DHB	2,6-DHB	3,4-DHB	3,5-DHB	3,4,5-THB
				**Gas phase**			
Aj	0.987	0.989	0.990	0.998	0.995	0.999	0.997
BAC	0.859	0.881	0.893	0.969	0.898	0.898	0.921
HOMA	0.944	0.949	0.956	0.983	0.975	0.986	0.980
GEO	0.030	0.025	0.022	0.003	0.011	0.002	0.008
EN	0.026	0.026	0.022	0.014	0.013	0.013	0.012
I6	90.91	91.83	92.31	97.17	94.41	97.96	95.41
NICS	-10.2616	-9.3056	-10.0787	-10.2780	-10.2731	-10.0251	-11.5274
				**Water solution**			
Aj	0.985	0.986	0.988	0.997	0.993	0.999	0.996
BAC	0.850	0.859	0.879	0.958	0.878	0.975	0.916
HOMA	0.936	0.936	0.948	0.974	0.967	0.983	0.975
GEO	0.033	0.032	0.026	0.006	0.016	0.001	0.008
EN	0.031	0.032	0.026	0.020	0.017	0.016	0.016
I6	90.42	90.63	91.58	95.92	93.32	98.54	95.16
NICS	-10.0741	-9.0564	-9.9075	-10.0358	-10.2185	-10.0103	-11.3553

Table S4. The distribution of electronic charges (calculated ChelpG and NBO) for dihydroxybenzoic acids and trihydroxybenzoic acid (gallic); Σē ring - total charge of the ring; Σē COO^- - total charge of the carboxylic anion

	**2,3-DHB**		**2,4-DHB**		**2,5-DHB**		**2,6-DHB**		**3,4-DHB**		**3,5-DHB**		**3,4,5-THB**	
	**NBO**	**ChelpG**	**NBO**	**ChelpG**	**NBO**	**ChelpG**	**NBO**	**ChelpG**	**NBO**	**ChelpG**	**NBO**	**ChelpG**	**NBO**	**ChelpG**
**Gas phase**														
C1	-0.206	-0.115	-0.245	-0.221	-0.198	-0.071	-0.234	-0.330	-0.175	-0.074	-0.127	0.024	-0.157	-0.049
C2	0.330	0.269	0.387	0.413	0.343	0.334	0.360	0.441	-0.189	-0.281	-0.234	-0.345	-0.210	-0.314
C3	0.245	0.259	-0.337	-0.440	-0.261	-0.288	-0.306	-0.388	0.273	0.365	0.332	0.544	0.285	0.344
C4	-0.244	-0.248	0.357	0.515	-0.200	-0.135	-0.157	0.056	0.274	0.255	-0.336	-0.544	0.231	0.135
C5	-0.219	-0.082	-0.314	-0.384	0.284	0.382	-0.307	-0.382	-0.270	-0.269	0.331	0.544	0.259	0.287
C6	-0.168	-0.178	-0.119	-0.001	-0.222	-0.322	0.358	0.458	-0.170	-0.096	-0.242	-0.351	-0.256	-0.328
**Σē ring**	**-0.262**	**-0.095**	**-0,271**	**-0.118**	**-0.254**	**-0.100**	**-0.286**	**-0.145**	**-0.257**	**-0.100**	**-0.276**	**-0.128**	**0.152**	**0.075**
C7	0.785	0.763	0.784	0.775	0.784	0.746	0.815	0.992	0.789	0.772	0.791	0.767	0.788	0.796
O1	-0.577	-0.555	-0.584	-0.560	-0.574	-0.545	-0.571	-0.625	-0.603	-0.596	-0.595	-0.651	-0.600	-0.601
O2	-0.702	-0.634	-0.708	-0.664	-0.707	-0.638	-0.672	-0.639	-0.694	-0.650	-0.687	-0.587	-0.698	-0.650
**Σē COO^-**	**-0.494**	**-0.426**	**-0.508**	**-0.449**	**-0.497**	**-0.437**	**-0.428**	**-0.272**	**-0.508**	**-0.474**	**-0.491**	**-0.471**	**-0.510**	**-0.455**
O3	-0.635	-0.528	-0.632	-0.535	-0.640	-0.557	-0.662	-0.615	-0.668	-0.606	-0.665	-0.644	-0.665	-0.575
O4	-0.707	-0.647	-0.662	-0.611	-0.677	-0.632	-0.665	-0.633	-0.698	-0.636	-0.665	-0.634	-0.695	-0.585
O5													-0.705	-0.625
**Water solution**														
C1	-0.219	-0.164	-0.259	-0.278	-0.210	-0.120	-0.260	-0.401	-0.186	-0.100	-0.137	0.001	-0.166	-0.074
C2	0.326	0.295	0.385	0.461	0.342	0.382	0.359	0.460	-0.202	-0.289	-0.245	-0.366	-0.224	-0.325
C3	0.252	0.267	-0.338	-0.472	-0.258	-0.308	-0.307	-0.395	0.267	0.360	0.331	0.558	0.278	0.346
C4	-0.234	-0.238	0.362	0.546	-0.201	-0.141	-0.154	0.057	0.287	0.280	-0.321	-0.547	0.238	0.136
C5	-0.220	-0.094	-0.308	-0.388	0.282	0.401	-0.307	-0.392	-0.265	-0.269	0.331	0.560	0.257	0.309
C6	-0.176	-0.181	-0.122	-0.004	-0.221	-0.332	0.358	0.484	-0.169	-0.103	-0.250	-0.363	-0.249	-0.332
**Σē ring**	**-0.271**	**-0.115**	**-0.280**	**-0.135**	**-0.266**	**-0.188**	**-0.311**	**-0.187**	**-0.268**	**-0.212**	**-0.291**	**-0.157**	**0.134**	**0.060**
C7	0.799	0.824	0.795	0.832	0.797	0.804	0.824	1.031	0.799	0.815	0.803	0.814	0.800	0.840
O1	-0.633	-0.637	-0.642	-0.645	-0.630	-0.630	-0.620	-0.690	-0.650	-0.664	-0.638	-0.650	-0.648	-0.669
O2	-0.697	-0.650	-0.704	-0.676	-0.700	-0.650	-0.676	-0.664	-0.692	-0.665	-0.686	-0.663	-0.691	-0.664
**Σē COO^-**	-0.531	-0.463	-0.551	-0.489	-0.533	-0.476	-0.472	-0.323	-0.543	-0.514	-0.521	-0.499	-0.539	-0.493
O3	-0.664	-0.587	-0.661	-0.604	-0.668	-0.622	-0.684	-0.656	-0.689	-0.643	-0.685	-0.679	-0.687	-0.614
O4	-0.713	-0.662	-0.675	-0.652	-0.696	-0.681	-0.684	-0.675	-0.698	-0.645	-0.686	-0.686	-0.696	-0.586
O5													-0.705	-0.635

Table S5. Wavenumbers [cm^{-1}], intensities and assignments of bands occurring in the experimental FT-IR of hydroxybenzoic acids.

**2,3-DHB**	**2,4-DHB**	**2,5-DHB**	**2,6-DHB**	**3,4-DHB**	**3,5-DHB**	**3,4,5-THB**	**Assignment**	**No. of the aromatic ring vibrations** [110]
3375 s	3374 s	3313 s	3414 m	3338 s	3214 vs	3368 vs	ν(OH)	
3048 m	3031 m	2924 m	3048 m	2966 m	3007 m	3013 m	ν(CH)	20b
2861-2578	2861-2555	2878-2578	2824-2545	2851-2585	2841-2504	2845-2574	ν(OH)	
1676 s	1639 vs	1669 vs	1676 vs	1671 vs	1688 vs	1704 vs	ν(C=O)	
		1619 s	1630 s	1602 vs	1609 vs	1619 s	ν(CC)	8b
1599 m		1603 sh	1579 m				ν(CC)	8a
	1521 m	1500 m		1525 m	1510 m	1541 m	ν(CC)	19b
1474 vs	1448 s	1445 vs	1474 s	1443 s	1480 s		ν(CC)	19a
1433 m	1409 m	1385 m	1419 s	1419 s	1416 s	1446 s	β(OH)	
1353 m	1348 m	1317 w	1354 m	1341 sh	1334 vs	1340 vs	ν(CC)	14
1302 s	1280 m	1278 m	1280 m		1305 s	1310 s	β(CH)	3
		1238 s	1229 m				ν(CH)	7a
1258 vs	1232 vs	1198 vs	1194 s	1283 vs	1263 m	1249 vs	νC-(OH)	
1235 s				1241 s	1208 m		β(OH)	
	1193 sh		1125 m			1207 m	ν(CH)	13
1159 s	1156 m		1160 m	1190 s	1164 vs		β(CH)	18a
1069 m	1091 m	1078 w	1068 w	1122 m	1109 sh	1102 w	β(CH)	18b
943 w	976 m	931 m	1031 m	942 m	1007 s	1025 s	ν(CH)	7b
	881 m	861 m			916 m	891 sh	γ(OH)	
831 w	848 m	844 m	809 s	890 w	851 m	865 m	γ(CH)	11
795 w	775 m	795 s	768 m	769 m	766 m	765 w	α(CCC)	12
746 s	693 m	755 m	724 sh		728 s		β(C=O)	
686 m	620 m	725 m	691 s	642 m	698 m	703 m	γ(C=O)	
633 m	603 sh		588 m	605 sh	623 w	576 m	γ(OH)	
507 m	531 w	555 m	533 w	558 w	565 w		α(CCC)	6a
	480 w				531 vw		φ(CC)	16b
453 m	449 w	470 m	459 w	452 m	471 m	442 w	ν CCC)	6b
419 w		415 w	424 w				β(CH)	9b

* fundamental modes of the phenyl ring are numbered according to Varsányi [110]; s – strong; m – medium; w – weak; v – very; sh – shoulder; ν: stretching; : in-plane deformations; : out of plane deformations; α: the aromatic ring in-plane bending modes; φ: the aromatic ring out-of-plane ones

Table S6. Chemical shifts δ [ppm] from the ^{1}H and ^{13}C NMR spectra of hydroxybenzoic acids.

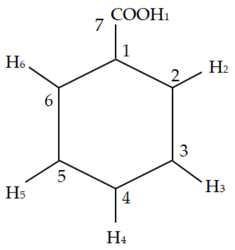

No.	2,3-DHB	2,4-DHB	2,5-DHB	2,6-DHB	3,4-DHB	3,5-DHB [114]	3,4,5-THB
δ_H							
1 (COOH)	11.40	11.42	10.70	10.48	12.28	12.60	12.20
2	9.28	10.30	9.15	10.48	7.34	6.85	6.91
3	9.28	6.27	6.78	6.34	9.64	9.60	9.17
4	7.01	10.30	6.96	7.22	9.28	6.46	8.86
5	6.71	6.34	9.15	6.34	6.78	9.60	9.17
6	7.24	7.62	7.17	10.48	7.29	6.85	6.91
δ_C							
1	113.18	107.97	112.69	102.32	121.71	132.48	120.46
2	150.44	164.05	154.22	160.62	116.61	107.31	108.73
3	145.95	102.30	114.67	106.34	144.94	158.35	145.42
4	120.00	163.42	123.85	134.67	150.06	106.81	138.00
5	118.61	104.38	149.48	106.94	115.22	158.35	145.42
6	120.76	131.91	117.86	160.62	121.97	107.31	108.73
7 (COOH)	172.49	171.90	171.82	172.36	167.45	167.31	167.48

Table S7. Perason correlation coefficient matrix between lipophilic, electronic parameters, aromaticity indices, FT-IR and NMR parameters of the selected dihydroxybenzoates. The determined correlation coefficients are significant with p <0.05000 N = 7 (missing data were removed by case).

Variable	LogP$_{C18}$	LogP$_{Galas}$	LogP$_{exp}$	pK$_{a1}$	ΔE $_{(LUMO-HOMO)}$	IP	BAC	HOMA	I6	NICS	NBO	νC-(OH)	β(CH) 18b	γ(CH) 11	δC2	δC3
LogP$_{Galas}$	0.8820															
	p=0.009															
LogP$_{exp}$	0.7741	0.9653														
	p=0.041	p=0.000														
pK$_{a1}$	-0.7842	-0.9186	-0.9177													
	p=0.037	p=0.003	p=0.004													
ΔE $_{(LUMO-HOMO)}$	0.7318	0.6250	0.4831	-0.5346												
	p=0.062	p=0.133	p=0.272	p=0.216												
IP	0.7954	0.5273	0.3621	-0.3590	0.8234											
	p=0.032	p=0.224	p=0.425	p=0.429	p=0.023											
BAC	0.3066	0.3226	0.3932	-0.4727	0.5573	0.2295										
	p=0.504	p=0.480	p=0.383	p=0.284	p=0.194	p=0.621										
HOMA	-0.0288	-0.2700	-0.2367	0.1225	0.2661	0.1845	0.7359									
	p=0.951	p=0.558	p=0.609	p=0.794	p=0.564	p=0.692	p=0.059									
I6	0.1617	-0.1256	-0.1199	-0.0179	0.3750	0.3725	0.7293	0.9672								
	p=0.729	p=0.788	p=0.798	p=0.970	p=0.407	p=0.411	p=0.063	p=0.000								
NICS	0.6804	0.5767	0.4717	-0.2650	0.3066	0.6373	-0.3480	-0.4585	-0.3079							
	p=0.093	p=0.175	p=0.285	p=0.566	p=0.504	p=0.124	p=0.444	p=0.301	p=0.502							
NBO	-0.6200	-0.3083	-0.0597	0.1685	-0.7059	-0.7746	0.0216	-0.0570	-0.1757	-0.4795						
	p=0.137	p=0.501	p=0.899	p=0.718	p=0.076	p=0.041	p=0.963	p=0.903	p=0.706	p=0.276						
νC-(OH)	-0.4738	-0.7437	-0.8806	0.8057	-0.2218	-0.0945	-0.4870	0.1016	0.0168	-0.1586	-0.3588					
	p=0.283	p=0.055	p=0.009	p=0.029	p=0.633	p=0.840	p=0.268	p=0.828	p=0.972	p=0.734	p=0.429					
β(CH) 18b	-0.4878	-0.7318	-0.7537	0.8650	-0.1656	-0.0281	-0.1077	0.4665	0.3420	-0.1467	-0.0724	0.7323				
	p=0.267	p=0.062	p=0.050	p=0.012	p=0.723	p=0.952	p=0.818	p=0.291	p=0.453	p=0.754	p=0.877	p=0.061				
γ(CH) 11	-0.7027	-0.7842	-0.7648	0.9254	-0.4300	-0.3708	-0.3211	0.1593	-0.0285	-0.2127	0.1593	0.7206	0.9035			
	p=0.078	p=0.037	p=0.045	p=0.003	p=0.336	p=0.413	p=0.483	p=0.733	p=0.952	p=0.647	p=0.733	p=0.068	p=0.005			
δC2	0.6404	0.8973	0.8873	-0.7858	0.3421	0.2514	-0.0108	-0.6424	-0.5338	0.5741	-0.0959	-0.7058	-0.8039	-0.6828		
	p=0.121	p=0.006	p=0.008	p=0.036	p=0.453	p=0.587	p=0.982	p=0.120	p=0.217	p=0.178	p=0.838	p=0.076	p=0.029	p=0.091		
δC3	-0.5492	-0.8310	-0.8866	0.6771	-0.4514	-0.2943	-0.3046	0.3280	0.2696	-0.4692	-0.0707	0.8090	0.5439	0.4877	-0.8545	
	p=0.202	p=0.021	p=0.008	p=0.095	p=0.309	p=0.522	p=0.507	p=0.473	p=0.559	p=0.288	p=0.880	p=0.028	p=0.207	p=0.267	p=0.014	

Table S8. PCA factor loadings, eigenvalues and explained percentage of variance.

	**PC1**	**PC2**	**PC3**
pK_{a1}	0.94	-0.05	0.25
γ(CH) 11	0.84	-0.01	0.21
δC3	0.82	0.19	0.12
νC-(OH)	0.78	0.15	0.54
β(CH) 18b	0.78	0.38	0.27
NBO (Σē ring)	0.27	-0.50	-0.74
HOMA	0.26	0.88	-0.38
I6	0.10	0.92	-0.29
BAC	-0.33	0.68	-0.61
IP	-0.53	0.60	0.53
NICS	-0.54	-0.12	0.71
$\Delta E_{(LUMO\text{-}HOMO)}$	-0.62	0.64	0.23
$LogP_{C18}$	-0.87	0.33	0.29
δC2	-0.89	-0.42	0.08
$LogP_{exp.}$	-0.96	-0.08	-0.17
$LogP_{Galas}$	-0.99	-0.00	0.04
Eigenvalue	8.16	3.61	2.61
Explained variance	51.0%	22.6%	16.3%
Cumulative eigenvalue	8.16	11.77	14.38
Cumulative explained variance	51.0%	73.6%	89.9%
